# Uncovering the Daily Experiences of People Living With Advanced Cancer Using an Experience Sampling Method Questionnaire: Development, Content Validation, and Optimization Study

**DOI:** 10.2196/57510

**Published:** 2024-11-05

**Authors:** Joran Geeraerts, Lara Pivodic, Lise Rosquin, Eline Naert, Geert Crombez, Mark De Ridder, Lieve Van den Block

**Affiliations:** 1 End-of-Life Care Research Group Vrije Universiteit Brussel Brussels Belgium; 2 Department of Medical Oncology Universitair Ziekenhuis Gent Ghent Belgium; 3 Department of Experimental Clinical and Health Psychology Universiteit Gent Ghent Belgium; 4 Translational Radiation Oncology, Physics and Supportive Care Research Group Vrije Universiteit Brussel Brussels Belgium

**Keywords:** cancer, quality of life, ecological momentary assessment, experience sampling method, telemedicine, mHealth, eHealth, patient outcome assessment, validated instruments

## Abstract

**Background:**

The experience sampling method (ESM), a self-report method that typically uses multiple assessments per day, can provide detailed knowledge of the daily experiences of people with cancer, potentially informing oncological care. The use of the ESM among people with advanced cancer is limited, and no validated ESM questionnaires have been developed specifically for oncology.

**Objective:**

This study aims to develop, content validate, and optimize the digital Experience Sampling Method for People Living With Advanced Cancer (ESM-AC) questionnaire, covering multidimensional domains and contextual factors.

**Methods:**

A 3-round mixed methods study was designed in accordance with the Consensus-Based Standards for the Selection of Health Measurement Instruments (COSMIN) and the European Organization for Research and Treatment of Cancer guidelines. The study included semistructured interviews with 43 people with stage IV breast cancer or stage III to IV lung cancer and 8 health care professionals. Round 1 assessed the appropriateness, relative importance, relevance, and comprehensiveness of an initial set of ESM items that were developed based on the existing questionnaires. Round 2 tested the comprehensibility of ESM items. Round 3 tested the usability of the digital ESM-AC questionnaire using the m-Path app. Analyses included descriptive statistics and qualitative content analysis.

**Results:**

Following the first round, we developed an initial core set of 68 items (to be used with all patients) and a supplementary set (optional; patients select items), both covering physical, psychological, social, spiritual-existential, and global well-being domains and concurrent contexts in which experiences occur. We categorized items to be assessed multiple times per day as momentary items (eg, “At this moment, I feel tired”), once a day in the morning as morning items (eg, “Last night, I slept well”), or once a day in the evening as evening items (eg, “Today, I felt hopeful”). We used participants’ evaluations to optimize the questionnaire items, the digital app, and its onboarding manual. This resulted in the ESM-AC questionnaire, which comprised a digital core questionnaire containing 31 momentary items, 2 morning items, and 7 evening items and a supplementary set containing 39 items. Participants largely rated the digital questionnaire as “easy to use,” with an average score of 4.5 (SD 0.5) on a scale from 1 (“completely disagree”) to 5 (“completely agree”).

**Conclusions:**

We developed the ESM-AC questionnaire, a content-validated digital questionnaire for people with advanced breast or lung cancer. It showed good usability when administered on smartphone devices. Future research should evaluate the potential of this ESM tool to uncover daily experiences of people with advanced breast or lung cancer, explore its clinical utility, and extend its validation to other populations with advanced diseases.

## Introduction

### Background

Quality of life assessment among people with cancer often relies on retrospective patient-reported outcome measures (PROMs), which typically require patients to aggregate their experience over several days or weeks into 1 score (eg, “During the past week, were you tired?”) [1-3]. This precludes temporally fine-grained knowledge on how cancer-related experiences such as physical or psychological symptoms and concerns change within and across days and the mechanisms underlying these changes. Moreover, studies found that retrospective PROMs often over- or underestimate in-the-moment somatic and psychological experiences across various populations, indicating a need for more fine-grained measures [4,5]. From a research and clinical perspective, this detailed knowledge on in-the-moment experiences is critical for improving patient symptom management and psychosocial support, such as by identifying novel intervention targets.

To bridge this gap, the experience sampling method (ESM) [6], also called ecological momentary assessments [7], may be suitable. The ESM or ecologic momentary assessments involve repeatedly gathering self-reported data from participants in the context of their daily lives, often multiple times per day for several consecutive days through mobile devices such as smartphones [7-9]. Contrary to traditional PROMs, the ESM mitigates retrospective biases and improves ecological validity of findings by asking questions about momentary experiences in their natural environment (eg, “At this moment, I feel...”) [7]. Moreover, the ESM provides the opportunity to study affect over time (ie, experiences of feelings or emotions) as an important indicator of emotional functioning and psychological well-being [9-11] and to investigate patients’ experiences together with concurrent contexts, such as the social environment [12]. Including contextual items can facilitate the identification of situations that alleviate or exacerbate certain experiences, thereby informing future psychosocial interventions.

Despite the ESM’s potential to provide novel insights into the daily experiences of people with cancer, its use in oncology research remains limited, especially among people with advanced (ie, metastatic) cancer [9,12,13]. Nevertheless, compared to people in the earlier stages of cancer, people with advanced cancer have a higher likelihood of experiencing symptoms and concerns that negatively impact their quality of life [14,15]. A possible explanation for the limited use of these methods among people with advanced cancer is that researchers may avoid them to prevent placing additional burden on patients through repeated assessments. However, to develop and improve interventions to alleviate these high levels of symptoms and distress, gaining a more detailed understanding of the well-being of people with advanced cancer in the context of their daily life (ie, its fluctuations, mechanisms, determinants, and consequences) is imperative; for this purpose, the use of the ESM is recommended [16,17]. The limited number of ESM studies among people with advanced cancer have investigated a range of symptoms, concerns, and measures of well-being across quality of life domains and provided evidence for the dynamic nature and associations thereof [18-30]. For example, Badr et al [21] found that greater pain in the morning was associated with feeling less aroused mood (eg, more tiredness and less peppy) during the rest of the day for women with metastatic breast cancer, with pain and low arousal mood being associated with romantic relationship interference.

There is currently no validated ESM questionnaire designed specifically for people with advanced cancer [9,13]. Validity, especially content validity, is a crucial indicator of whether the content of an instrument is an adequate reflection of the construct being measured [31,32]. However, it is often overlooked in ESM research as a whole, leading to recent calls for more content validation of ESM questionnaires [9,13,32,33].

By reporting the development, content validation, and optimization of an ESM questionnaire, this study is the first step of a larger project in which we aim to test the feasibility of the ESM and use it to obtain novel insights into the daily experiences of people with advanced cancer. Because symptoms can vary across different advanced cancer diagnoses and our aim was to develop a questionnaire that is highly relevant to the specific experiences of intended users, our project’s scope is narrowed to people living with advanced breast or lung cancer. We selected these diagnoses as they are among the most prevalent cancer diagnoses with high mortality rates [34-36] and are associated with considerable risk for experiencing serious symptom burden [37-41].

### Objectives

In this study, we aimed to develop, validate, and optimize the Experience Sampling Method for People Living With Advanced Cancer (ESM-AC) questionnaire. The digital ESM questionnaire aims to comprehensively assess relevant daily experiences (ie, symptoms, concerns, and well-being) of people with advanced breast or lung cancer and the context in which these experiences occur; it collects these data multiple times per day for several consecutive days.

## Methods

### Study Design

**Figure 1:**
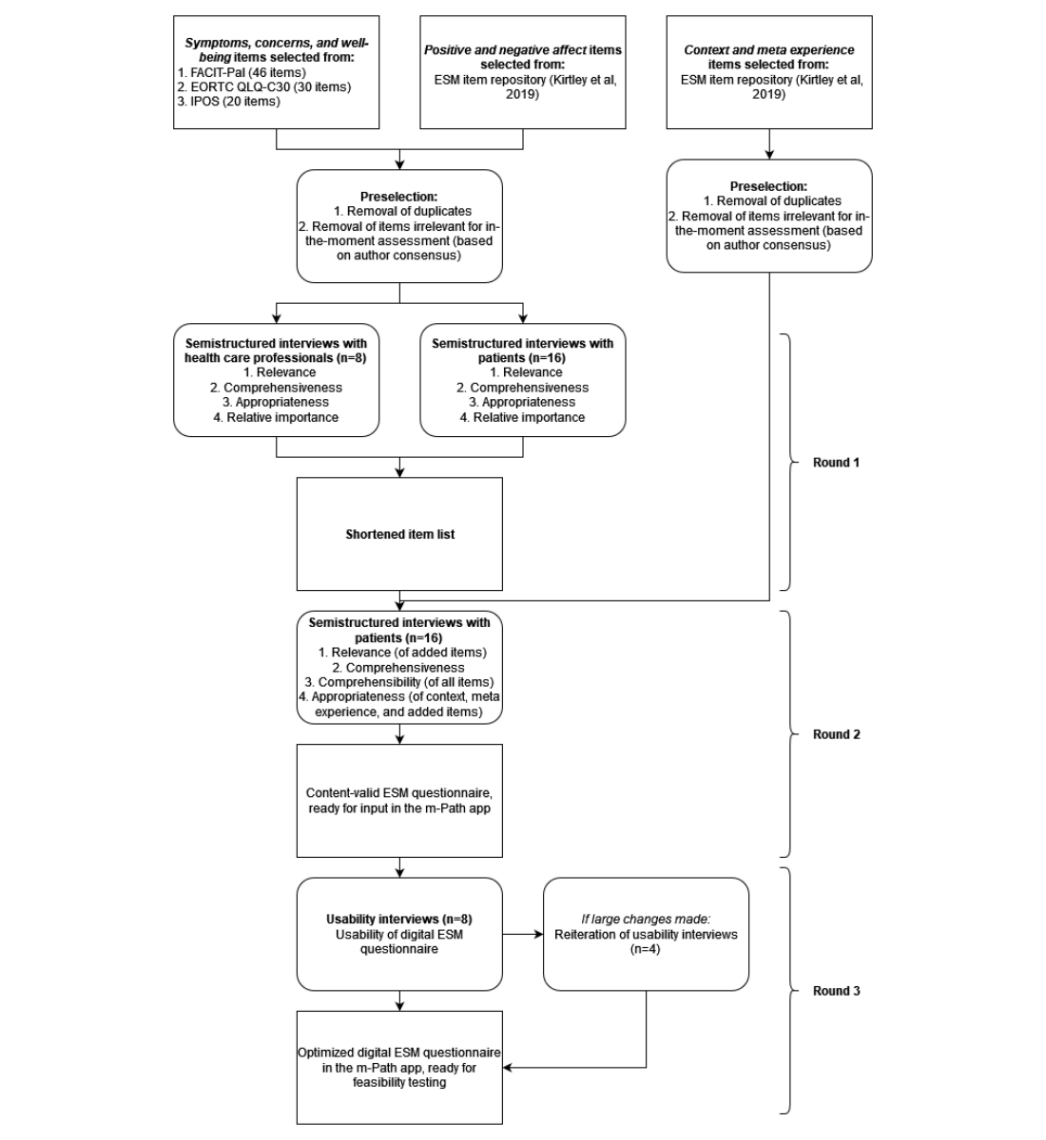
Flowchart of the development and validation procedure [42]. EORTC QLQ-C30: European Organization for Research and Treatment of Cancer Core Quality of Life Questionnaire; ESM: experience sampling method; FACIT-Pal: Functional Assessment of Chronic Illness Therapy—Palliative Care; IPOS: Integrated Palliative Care Outcome Scale.

We conducted a 3-round interview study with patients and health care professionals using a mixed methods research design (summarized in Figure 1 [42]). To develop and validate the ESM questionnaire in the first 2 interview rounds, we based our design on the guidelines of PROMs [31,43] because no specific guidelines for ESM questionnaires were available [32]. Specifically, the Consensus-Based Standards for the Selection of Health Measurement Instruments (COSMIN) methodology [31] guided the assessment of the content validity of our initial set of items in the first 2 rounds (ie, covering relevance, comprehensibility, and comprehensiveness; refer to Textbox 1 for an overview of key psychometric concepts used in this study). In the first round, the item set was shortened and categorized into a core and supplementary item set based on content validity, appropriateness, and relative importance, following the European Organization for Research and Treatment of Cancer (EORTC) guidelines for module development [43-45]. The second round focused on the comprehensibility of all items and on the relevance and appropriateness of the items added after round 1. In the third round, we optimized the digital (core) ESM questionnaire by assessing barriers related to its usability for patients using the dedicated ESM smartphone app (ie, m-Path; KU Leuven [46]).

**Key concepts with their respective definitions.**

- Content validity: the extent to which the content of an instrument is an adequate reflection of the construct to be measured. This includes relevance, comprehensiveness, and comprehensibility [31].
- Relevance: the extent to which a questionnaire item is relevant for the construct of interest within a specific population and context of use [31].
- Comprehensiveness: the extent to which all key aspects of the construct are included in the questionnaire [31].
- Comprehensibility: the extent to which a questionnaire item is understood by patients as intended [31].
- Appropriateness: the extent to which a questionnaire item is perceived as appropriate and not upsetting [43].
- Relative importance: the extent to which a questionnaire item is deemed more important for the questionnaire’s context of use than other items in the same content domain.
- Usability: the extent to which a system, product, or service can be used by specified users to achieve specified goals with effectiveness, efficiency, and satisfaction in a specified context of use [47].

### Ethical Considerations

This study was approved by the central ethics committee of university hospital Brussels (Belgian Unique Numbers: 1432021000533 and 1432023000043) and by the local committee of general hospital Aalst, Belgium. All participants provided written informed consent before study participation. Patients did not receive any compensation. Health care professionals received a €25 (US $27.06) gift card. Data were treated confidentially and were strictly analyzed in a deidentified form.

### Participants and Setting

For the first 2 rounds, we planned to interview 32 patients and 8 health care professionals from 1 university hospital and 1 regional hospital in Belgium. These sample sizes adhere to the COSMIN and EORTC guidelines [31,43]. In the third round, we aimed to include 8 patients from the former university hospital [48] and 4 additional patients if, after the previous usability interviews, large changes would be made that would require further testing. JG and the hospital staff identified eligible patients through clinic appointment lists, and JG invited patients to participate via telephone or in-person communication during hospital visits. Health care professionals were identified through the research team’s professional networks and contacted via email.

Inclusion criteria included the following: (1) a diagnosis of stage III or IV lung cancer or stage IV breast cancer; (2) patient aged ≥18 years; (3) patient spoke and understood the Dutch language; and (4) patient assigned an Eastern Cooperative Oncology Group performance status of 0, 1, or 2, based on the assessment by their treating physician.

Exclusion criteria included the following: (1) patient having major communication difficulties or insufficient cognitive abilities to take part in a semistructured interview (as judged by their treating physician); (2) patient having any psychiatric disorder that, in the opinion of their treating physician, might hinder participation due to expected burden or unreliable responses; (3) patient having uncorrectable hearing or poor vision; or (4) patient had participated in a previous part of this study.

We aimed to include 4 equally sized subgroups based on the primary tumor site (breast or lung cancer) and age (<70 years or ≥70 years) [49,50].

As for health care professionals, we aimed to include a specialist in respiratory oncology, an oncologist specialized in breast cancer, a radiotherapy specialist, an oncology nurse, an onco-psychologist, a health sciences researcher, and 2 specialist palliative care providers (ie, a physician and a nurse affiliated with a palliative home care team).

### Measurement Instruments and Procedures

#### Initial Item Set

**Figure 2:**
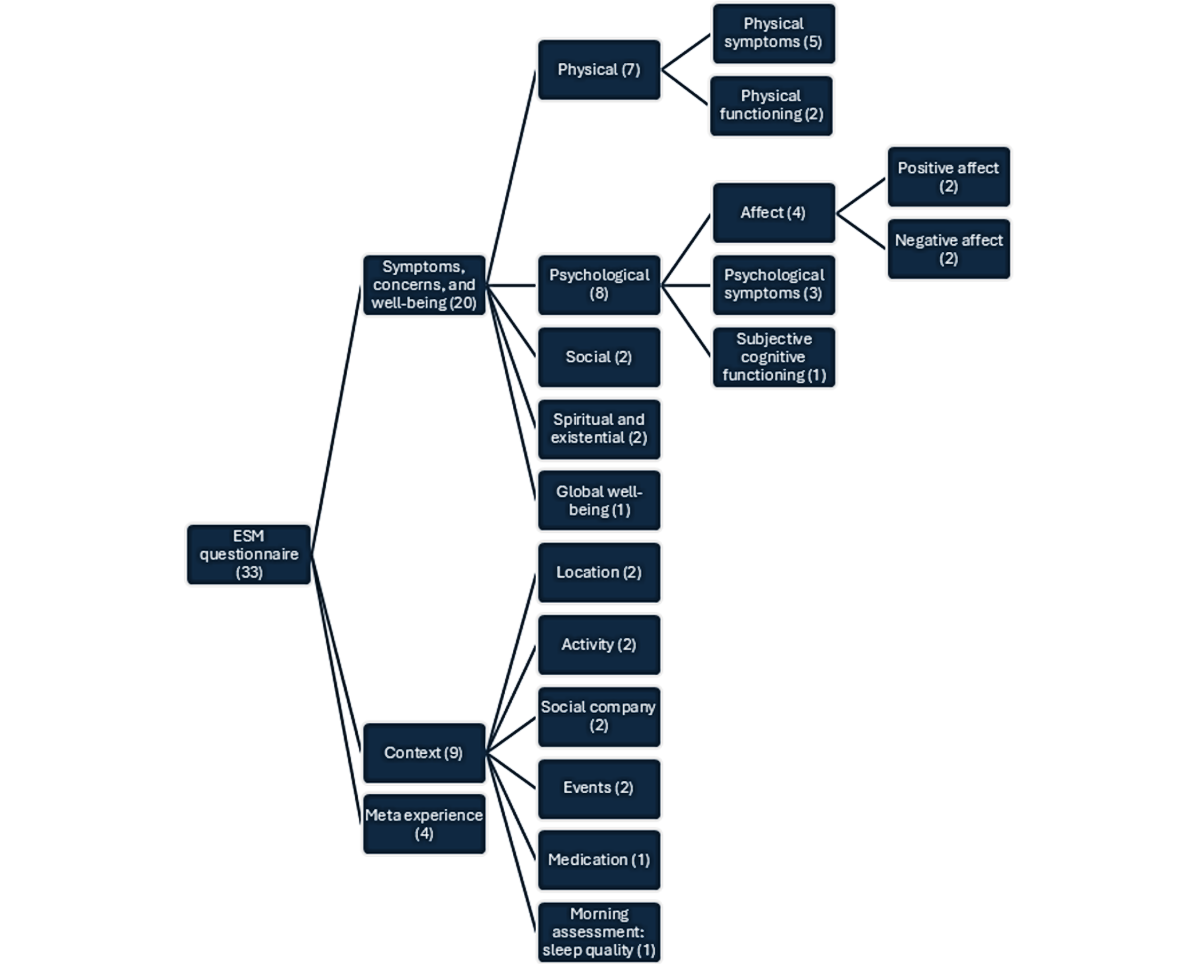
Subdomains that the Experience Sampling Method for People Living With Advanced Cancer (ESM-AC) questionnaire intended to cover. Note that the between-bracket numbers after each domain name indicate the approximate number of items that we aimed to retain per domain and the number of most important items that participants had to choose for each right-most subdomain.

The questionnaire aimed to comprehensively measure and evaluate daily experiences of people with advanced cancer and the context in which they occur. More specifically, we conceptualized daily experiences as symptoms, concerns, and well-being across physical (including physical symptoms and functioning), psychological (including positive and negative affect, psychological symptoms, and cognitive concerns), social, spiritual-existential, and global well-being domains (Figure 2). Context was conceptualized as the person’s current location, activity, social company, substantial events, medication use, and sleep quality.

We created an initial item set capturing in-the-moment experiences based on (1) the items of questionnaires identified in the 2018 review of PROMs in patients with advanced cancer by van Roij et al [1] and (2) an existing ESM item repository from the field of mental health sciences [42]. From the review by van Roij et al [1], we selected 3 questionnaires: the European Organization for Research and Treatment of Cancer Core Quality of Life Questionnaire (EORTC QLQ-C30), Functional Assessment of Chronic Illness Therapy—Palliative Care (FACIT-Pal), and the Integrated Palliative Care Outcome Scale [51-53], as they relate to our target population, have sufficient content validity, and have a comprehensive symptom coverage (ie, did not focus on one specific symptom or experience). On the basis of the consensus achieved through discussion among the authors, we excluded overlapping items and items with low expected intraday variability (eg, “I have family members who will take on my responsibilities”) and retained 43 items suitable for the measurement of symptoms, concerns, and well-being across various subdomains (Figure 2). When consensus was required for adding, changing, or removing items, the content was first discussed primarily among JG, LP, and LVdB, who are all trained psychologists. LP and LVdB have >10 and 20 years of experience as end-of-life researchers, respectively. JG had 1 year of prior expertise in ESM mental health research. If further discussion or advice was needed, other authors were consulted, including a research assistant (LR; no prior expertise), a medical oncologist (EN; 7 years of experience), a health psychology researcher with experience in ESM research (GC; ≥30 years of experience), and a radiation oncologist (MDR; ≥20 years of experience).

From the ESM item repository, we purposively selected 12 items measuring affect, spanning across the valence and arousal dimensions [54] (ie, levels of pleasantness and physiological activation, respectively), and 13 items measuring context. We additionally selected items to measure the patient’s experience while completing the ESM questionnaire (ie, meta-experience items). We obtained official Dutch translations for all items and rephrased them to reflect in-the-moment experiences (eg, changing “During the past 7 days, I felt...” to “In this moment, I feel...” or “Since the last beep, I felt...” with “beep” referring to the assessment prompt). For less frequent experiences or events, such as, for the item “I have had diarrhea,” we used the phrase “Since the last beep” instead of “In this moment.” One item measuring sleep quality was adapted from the FACIT-Pal questionnaire [52] and used for the first assessment of the day (ie, “Last night, I slept well”). All English translations of items presented in this paper are phrased analogous to their existing PROM counterparts, or if no such counterparts were available, we provided translations of the Dutch versions used in this study.

#### Content Validity and Usability Assessments

In all study rounds, we conducted individual semistructured interviews with patients with advanced breast or lung cancer. One round also included interviews with health care professionals, as outlined in Figure 1. These interviews served to assess content validity, to shorten the initial item list and divide it into a core and supplementary set, and to optimize the digital ESM questionnaire based on its usability. At the start of all interviews, the patients completed a baseline questionnaire on sociodemographic and clinical characteristics (age, gender, living situation, marital status, education level, employment status, religious denomination, and received treatments). In round 3, the baseline questionnaire additionally assessed cognitive concerns [55] and smartphone use [56,57]. We conducted all interviews in person, either at patients’ homes or in quiet hospital rooms. Patients’ friends and relatives were allowed to be present during the interviews. Across rounds, we introduced the ESM to participants as a digital diary on a smartphone device that uses 10 assessments per day for several consecutive days to study people’s symptoms, concerns, well-being, and daily situations as well as their fluctuations within and across days.

During round 1, JG interviewed patients and health care professionals to evaluate the relevance and comprehensiveness of symptoms, concerns, and well-being items. We aimed to create a core item set of 33 items, which was the foreseen number of items needed to cover all subdomains, and a supplementary set with no item limit and aimed to improve its comprehensiveness by adding items deemed relevant but missing by the participants. Participants were asked to verbally rate each item’s relevance (“not at all,” “a little,” “quite a bit,” and “very much”), select the most important items for each subdomain (Figure 2 displays the number of items to select per subdomain, as instructed by the interviewer), suggest missing concepts, and mark inappropriate items. Participants were prompted for reasons for categorizing items as inappropriate or “not at all” or “a little” relevant.

In round 2, JG interviewed patients on the comprehensibility of items resulting from the first round (as the last part of content validation), the relevance and appropriateness of newly added items, and the appropriateness and comprehensiveness of context and meta-experience items and their response options (assessed analogous to round 1). To assess comprehensibility, patients completed a pen-and-paper questionnaire while thinking out loud [58].

In round 3, JG and LR conducted interviews to assess and optimize the ESM questionnaire’s usability by letting patients respond to it in the m-Path app [46]. m-Path is a web-based platform that provides “an intuitive and flexible framework to conduct smartphone-based ecological momentary assessment and intervention studies...” [46]. Patients were each provided with a Motorola E20 smartphone device (Motorola Mobility LLC) with the digital ESM questionnaire available in the m-Path app. They were instructed on how to use the app and asked to complete the digital questionnaire on the provided device while thinking out loud. The researcher prompted patients when difficulties were observed (eg, difficulties answering certain ESM questions). Afterward, a brief semistructured interview assessed the usability of the questionnaire through an adapted version of the System Usability Scale (5-point Likert scale; 1=totally do not agree and 5=totally agree) [59,60]. Usability outcomes included readability, comprehensibility, ease of use, reasons for encountered difficulties, and expected burden of receiving 10 assessments per day for 6 days. Finally, patients completed the digital ESM questionnaire a second time without thinking out loud to estimate completion times. All interviews were recorded and transcribed verbatim. More details on procedure and instruments for this round have been reported in the study protocol [61].

### Data Analyses and Continuous Adaptations of the Questionnaire

Following the EORTC guidelines for module development, as applied by Sprangers et al [43] and Groenvold et al [45], we transformed item relevance ratings into a 0 to 100 scale, with “not at all” corresponding to 0 and “very much” to 100. We calculated mean relevance scores and SDs per item. In addition, we calculated the percentages of respondents who rated an item as inappropriate or upsetting, who listed an item among the top *n* most important items per subdomain (*n* was the approximated number of items to retain in the final questionnaire for each subdomain; Figure 2), and who found an item incomprehensible. We calculated descriptive statistics for usability.

Using conventional content analysis [62] on the interview transcriptions, we inductively developed content categories for participants’ reasons of lack of item relevance (provided by participants who judged an item as “not at all” or “a little” relevant), inappropriateness, problems with comprehensibility, and themes of novel items to add [62]. We added items to the list if at least 2 participants suggested adding it to the questionnaire. Furthermore, we developed content categories for difficulties or conveniences in the user experience or comprehension of the digital questionnaire.

The questionnaire was adapted after each of the 3 rounds. After round 1, we used descriptive statistics of relevance, importance, and appropriateness ratings from the patients and health care professionals to guide item exclusion and categorization into core and supplementary sets (refer to Multimedia Appendix 1 for an overview of the categorizations). We assigned items to the core item set if they ranked among the top *n* most important per subdomain (refer to Figure 2 for n values), were judged “quite a bit” or “very much” relevant by half of the participants (50%), and were deemed appropriate (or amenable to rewording). For the removal of items, the authors discussed the participants’ reasons for low relevance of items that were rated as “not at all” or “a little” relevant by at least half of the participants, or of items for which the participants provided recurring reasons for lack of relevance or the inappropriateness of items and the item could not be appropriately reworded or changed to resolve those reasons. Items that were not removed or categorized into the core set were assigned to the supplementary set. Note that the decision to use the core and supplementary sets was made after analysis of round 1.

After round 2, we made necessary and feasible item revisions based on the descriptive statistics of comprehensibility and inappropriateness and on the content categories for reasons of items’ low comprehensibility and inappropriateness.

After round 3, we used descriptive statistics of usability outcomes and content categories of difficulties when using the digital questionnaire to improve the usability of the questionnaire in m-Path. Following general recommendations in ESM research [16,63], we used a mean questionnaire completion time threshold of 3 minutes to determine whether the questionnaire was considered too long.

## Results

### Participant Characteristics

In round 1, a total of 15 patients and 8 health care professionals participated; in round 2, a total of 18 new patients participated; and in round 3, a total of 10 new patients participated (Table 1). The overall mean age was 67.3 (SD 10.3) years. Overall, 23 (53%) of the 43 patients had a stage III or IV lung cancer diagnosis, and the remaining 20 patients (47%) had a stage IV breast cancer diagnosis. Close others were present during 4 interviews in round 1, seven in round 2, and seven in round 3.

**Table 1:** Sociodemographic and clinical characteristics of patients per interview round (N=43).

Characteristics			Round 1 (n=15)^a^		Round 2 (n=18)^b^		Round 3 (n=10)
**Age (years)**							
	Mean (SD)	68.0 (8.5)		68.7 (11.3)		63.8 (11.1)	
	Range	56-78		44-86		45-78	
Gender (female), n %			11 (73)		14 (78)		6 (60)
**Living situation, n**							
	Living alone at home	2		4		2	
	Living with a partner/children/others at home	13		14		8	
**Marital status, n**							
	Married	13		8		—^c^	
	Living together but not married	0		6		—	
	Widowed	1		1		—	
	Divorced	1		3		—	
**Educational level, n**							
	Primary	2		0		1	
	Secondary	8		10		4	
	Tertiary	5		8		5	
**Employment status, n**							
	Professionally active	2		1		1	
	Not professionally active	13		17		9	
**Religious denomination, n**							
	Catholic Christian	6		8		6	
	Not religious	5		9		4	
	Not specified	4		1		0	
**Cancer diagnosis, n**							
	Stage III or IV lung cancer	7		10		6	
	Stage IV breast cancer	8		8		4	
**Treatment or treatments received, as reported by the patient, n**							
	Chemotherapy	14		13		9	
	Radiotherapy	13		10		5	
	Surgery	12		3		7	
	Hormonal therapy	4		5		2	
	Immunotherapy	6		9		4	
**EORTC QLQ-C30^d^ concentration problems, n**							
	Not at all	—		—		7	
	A little	—		—		2	
	Quite a bit	—		—		1	
	Very much	—		—		0	
**EORTC QLQ-C30 memory problems, n**							
	Not at all	—		—		5	
	A little	—		—		3	
	Quite a bit	—		—		2	
	Very much	—		—		0	
Smartphone ownership in years, mean (SD)			—		—		10.2 (4.4)
Daily time spent on smartphone in hours, mean (SD)			—		—		3.2 (2.8)
Confidence using smartphone (1=“not at all confident,” 5=“very confident”), mean (SD)			—		—		4.1 (0.7)

^a^Due to an oversight, we did not collect participation rates and reasons for nonparticipation in this round.

^b^Out of 25 invited patients. Reasons for nonparticipation included no interest, as indicated by patient or partner (n=5), inability to find an appropriate interview location (n=1), experiencing distress (n=1), or no reasons provided (n=1).

^c^Not measured.

^d^EORTC QLQ-C30: European Organization for Research and Treatment of Cancer Core Quality of Life Questionnaire.

The following sections present the results per interview round and relevant adaptations made to the ESM questionnaire based on these findings.

### Interview Round 1

#### Relevance

Most items received positive relevance ratings, with no unanimous low relevance ratings across all participants (Multimedia Appendix 2). The most frequent reasons for considering an item lacking in relevance were overlapping content with other items, not experiencing the measured construct, not perceiving the measured construct as bothersome, and thinking the item could be phrased better. After discussion among the research team, we removed 12 items that at least half of the participants rated as having “a little” relevance or less or that participants noted had considerable overlapping content with other items. For instance, we removed the item “At this moment, I feel sick” due to overlap with specific symptoms such as nausea and removed the item “At this moment, I feel capable of making decisions” due to low reported relevance because patients reported not having to make decisions.

Some items were considered irrelevant by the participants because they measured stable constructs within a day. To address this, we deviated from the planned approach to develop in-the-moment items only and instead developed several items for designated morning and evening assessments. We dedicated 1 item of the initial item list to morning assessments and 11 to evening assessments. For instance, the in-the-moment item “At this moment, I feel moral support by my close ones” was revised to the evening item “Today I felt supported by others.” Items excluded before round 1 based on little expected within-day variability were reconsidered for inclusion in the once-daily questionnaires. Hence, we added 8 initially removed items to the evening list for further testing in round 2 (eg, “Today, I was able to openly discuss my concerns with my close ones”).

#### Appropriateness

Out of 55 items, 22 (40%) were deemed inappropriate by between 1 and 5 participants (Multimedia Appendix 2), with 12 (22%) items deemed inappropriate by at least 2 participants. Reasons included privacy concerns, content overlap, confronting questions, infrequent experiences, question formulation, clinical utility, and bad subdomain fit (Multimedia Appendix 3). We removed the most inappropriate item “At this moment, I feel enthusiastic” as 4 patients and 1 health care professional marked it as inappropriate due to content overlap and patients not experiencing this feeling.

#### Comprehensiveness

Participants suggested adding several constructs to improve comprehensiveness, leading to the addition of 13 items to the item list (Multimedia Appendix 4). Among these, 2 were conditional items administered only if certain responses are given during the same assessment, such as reporting moderate pain levels or poor sleep. These questions included “The pain is located in these parts of the body: ...” and “I think I didn’t sleep so well, because: ... .” Examples of other added items included “At this moment, I feel capable of working” and “At this moment, I have negative thoughts or feelings.” In addition, we included 3 items in the questionnaire as the research team thought them to be necessary for comprehensive measurement of the psychological domain (“At this moment, I feel restless” and “At this moment, I feel depressed”) and an open question concerning other contextual factors (“If there is anything else you want to mention about the period since last beep, you can do that here:”).

#### Relative Importance

We assigned 46 items with the highest relative importance of their subdomain to the core questionnaire and 38 items to the supplementary list (refer to Multimedia Appendix 2 for the proportions of how many times items were chosen as among the top most important).

### Interview Round 2

#### Comprehensibility

Between 1 and 5 participants provided remarks for 31 (39%) out of 79 items (Multimedia Appendix 5). Reasons for marking items as incomprehensible included unclear word meanings, different interpretations from the intended meaning, situational content, response options misalignment, and other issues. In response to this feedback, we changed the wording of some items and response options and removed some items (Multimedia Appendix 6). For instance, we replaced the response option “On the move” under the item “What am I doing?” to “En route (eg, on the bus)” for clarity. Another example is the core questionnaire item “Today I felt supported by others,” which we changed to “Today I received the support I needed from my loved one(s)” because some patients indicated not needing or seeking support all the time.

#### Relevance of Added Items

On average, most added items were rated as at least “a little” relevant, with mean ratings typically exceeding “quite a bit” relevant (Multimedia Appendix 2).

#### Appropriateness of Added Items

No items were considered as inappropriate by the participants.

#### Additional Findings and Changes Made

**Figure 3:**
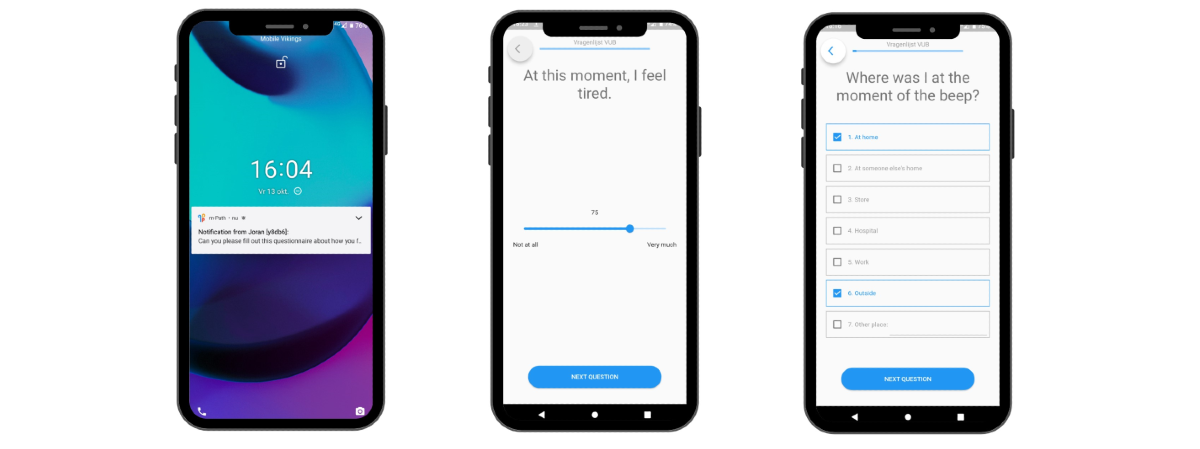
Screenshots of the Experience Sampling Method for People Living With Advanced Cancer questionnaire in the m-Path app. Left: receiving a notification, middle: example of the slider response scale; right: example of the multiple-choice response scale.

Three patients reported frequently experiencing muscle cramps, leading to the addition of the item “Since the last beep, I had muscle cramps” to the supplementary list. On the basis of research team consensus, we improved the comprehensiveness of the “Where am I?” item by adding an “outside” response option. Figure 3 displays the resulting questionnaire in the m-Path app.

### Interviews Round 3

#### Usability

On a scale ranging from 0=“completely disagree” to 5=“completely agree,” participants generally expressed positive sentiments about using the ESM-AC questionnaire in their daily lives (mean 3.6, SD 0.8), finding it easy to use (mean 4.5, SD 0.5), and expecting no need for support with the questionnaire or the smartphone device in their daily lives (mean 1.6, SD 0.7 and mean 1.5, SD 0.7, respectively). They also indicated that there was no inconsistency in the questionnaire (mean 1.6, SD 0.7). They expected that most people would quickly learn to use the questionnaire (mean 4.0, SD 1.1), felt confident using it (mean 4.2, SD 1), did not require a lot of knowledge to complete it (mean 1.3, SD 0.5), items and response options were clear (mean 4.3, SD 0.5 and mean 4.0, SD 0.9; respectively), the response options were comprehensive (mean 4.1, SD 1), and the lay-out was satisfying (mean 4.2, SD 0.6). Moreover, participants did not experience it as burdensome to complete the questionnaire (mean 1.5, SD 0.7) and did not think it was too long (mean 1.9, SD 0.9). However, as reflected by neutral mean scores with higher variance, participants were more divided regarding the simplicity of item phrasings (mean 2.2, SD 1.2) and the readability of items (mean 3.9, SD 1.4). Moreover, most participants anticipated that completing the questionnaire 10 times per day on 6 consecutive days would be burdensome (mean 3.7, SD 1.1).

#### Perceived Difficulties

Participants reported various barriers with using the digital ESM-AC questionnaire and device, and we observed some difficulties when participants used the questionnaire. For some patients, response formats and the option to skip open-ended items were initially not clear, the momentariness of items (ie, “At this moment, I feel...”) required further instructions (eg, participants would give higher pain scores due to previous pain episodes, when currently not experiencing pain), interpretations of some complex items were unintended (eg, concentration problems were interpreted as wider cognitive problems), the purpose of the intensive assessment schedule of the ESM study and of specific questionnaire content domains were unclear (eg, context items), and the device went into standby mode during the interview. All the changes made to the ESM-AC questionnaire, smartphone device settings, and onboarding instructions are reported in Table 2. Refer to Multimedia Appendix 7 for the resulting core ESM questionnaire. We also created a manual for researchers to provide patients with instructions where needed (Multimedia Appendix 8).

**Table 2:** Changes made to different ESM-AC^a^ questionnaire properties after the usability interviews of round 3.

Property and observed or reported barriers		Changes made
**ESM^b^ questionnaire**		
	Momentariness of item unclear	The phrasing “at the moment the beep went off” was added to the multiple-choice context items. For example: “Who am I with?” was replaced with “Who was with me at the moment of the beep?”
	Momentariness of item unclear	In-the-moment phrasings were added to items that did not previously include it. For example: “I’m in bed or on the couch” was replaced with “I was in bed or sofa when the beep went off.”
	Meaning of “place I was at” wrongly associated with bed or sofa	“I was happy with the place I was at” was reordered to be between “Where was I at the moment of the beep?” and “I was in bed or sofa when the beep went off.”
	Unclear what was measured with substance item	“Since last beep, I have used the following” was replaced with “Since last beep, I have used the following substance(s)”; the response option “Other” was changed to “Other substance(s).”
	Need for additional open-ended items when participants used the “Other” response option	An m-Path app feature was selected for the multiple-choice items that allows participants to directly type new categories when the “Other” option is selected.
**Smartphone device settings**		
	Device screen darkened while completing the questionnaire	The time-to-standby settings on the devices was changed from 30 seconds to 60 seconds.
**Onboarding instructions**		
	Response formats and option to skip open-ended items were not initially clear, momentariness of items required instructions, unintended interpretations of some complex items, purpose of the intensive assessment schedule of the ESM study and of some study domains (eg, context items) was unclear, reported expectations of missing assessments, and difficulty unlocking the smartphone	A formal interview guide was developed for the training at the onboarding session, which included instructions on how to explain the different response option formats and how to use them, skipping open-ended items, temporality of questions (ie, in-the-moment or since the last beep), content of more complex items (eg, concentration as separate from memory problems), the purpose of the intensive assessment schedule of the ESM study and of some question domains, acceptability of missing assessments, and unlocking the smartphone.

^a^ESM-AC: Experience Sampling Method for People Living With Advanced Cancer.

^b^ESM: experience sampling method.

#### Completion Times

During the second time of filling in the digital ESM-AC questionnaire (ie, without thinking out loud), it took participants on average 3.8 (SD 1.1) minutes to complete the questionnaire of 25 to 31 items (depending on the number of triggered conditional items).

## Discussion

### Principal Findings

We developed, content validated, and optimized the ESM-AC questionnaire, a digital ESM questionnaire covering multidimensional domains to capture the experiences of people with advanced breast or lung cancer. Overall, the patients found the questionnaire items comprehensible and appropriate and had positive views toward using the questionnaire in the m-Path app. As all items in the initial set were relevant to at least some patients, we primarily used the perceived importance of the items to categorize them into a core questionnaire for use with all patients and a supplementary item set from which patients can select items to tailor the ESM questionnaire to their needs and experiences.

As a novel and promising tool to assess patients’ symptoms, concerns, and overall well-being, the ESM-AC questionnaire supplements the existing measurement methods in oncology, a field that has traditionally relied on retrospective PROMs [1-3]. The ESM uniquely allows for the measurement of experiences in real time within the patient’s everyday life [16]. By using multiple assessments per day, it enables the investigation of how these experiences change and unfold over time, including their correlations and temporal relationships [16]. The repeated within-day assessments of the ESM can also supplement more traditional daily diary measures in oncology that assess patients once per day to uncover fine-grained fluctuations of symptoms. This can be important to better understand the complexity and dynamics of patient experiences from a research perspective. Moreover, from a clinical perspective, the ESM can be used to improve understanding of symptoms or concerns of individual patients identified using traditional once-daily or weekly administered PROMs.

To the best of our knowledge, the ESM-AC questionnaire is the first of its kind in oncology in several respects. First, the limited number of ESM studies in populations with cancer have never determined the content validity of their questionnaire items to be assessed in a repeated in-the-moment context [9,13]. Second, in cancer ESM research, the ESM-AC questionnaire is among the first to incorporate items on context and context appraisal [9,12]. By including items on concurrent location, activity, and social company, it will be possible to better understand fluctuating symptoms and their interactions with contextual factors. ESM research in other fields has shown how different contexts such as social company, concurrent activities, and location can influence patients’ mental and physical experiences [64-66]. Third, by dividing items into a core and supplementary list, item selection can be adapted or tailored to a particular patient or a population of patients, that is, by adding relevant supplementary items such as “At this moment, I feel capable of working.” This makes our ESM measurement highly relevant for people with advanced breast or lung cancer.

Using the m-Path app [46], results showed that the ESM-AC questionnaire was easy to use for all patients, and the patients had positive views toward the questionnaire presented on the device. This is crucial because it is important to minimize the potential burden of frequent daily assessments. This is especially true when working with populations that may be more likely to experience increased symptoms and reduced physical functioning related to cancer and related treatments. In addition, although the questionnaire took, on average, longer than the generally recommended 3 minutes’ completion time in ESM research [16,63], participants indicated that it was not too long. Therefore, we deviated from our initial 3-minute threshold and did not further shorten the questionnaire [61]. As we purposively sampled people aged >70 years and <70 years (mean 63.8, SD 11.1; range 45-78 years), we were able to conclude that the system questionnaire was usable for older age groups (ie, those aged ≤78 years) that are typically thought to have less smartphone experience, as indicated by their positive views on usability of the system.

### Implications for Future Research

The next step in the development of the ESM-AC questionnaire is to evaluate it in a detailed pilot ESM study. Such a study needs to evaluate the optimal number of daily assessments among people with advanced lung cancer or advanced breast cancer. As most participants indicated that they expected 10 assessments per day for 6 consecutive days, as is often used in ESM research [16], to be potentially burdensome, the burden of completing such an intensive assessment schedule should be carefully investigated in real life. This burden needs to be weighed against the necessary resolution to measure change in the construct of interest. In addition, further research is needed regarding the acceptability of the questionnaire length and clarity of the instructions, items, and response options if researcher help is not immediately available. If further research confirms the feasibility and optimal features for a larger-scale ESM study, this will pave the way toward a substantial improvement of our knowledge of how symptoms, concerns, and well-being across multiple domains fluctuate in the everyday life of people with advanced breast or lung cancer.

Researchers aspiring to apply similar methods to other populations with cancer or serious illness are encouraged to further adapt the methods to their target population. We recommend the ESM-AC questionnaire as a starting point for adaptations toward the target population and context. The core ESM questionnaire can be used in its entirety or researchers can select the domains of interest, possibly supplemented by items selected from the supplementary item set. Determining the questionnaire’s content validity through semistructured interviews will help to optimize and ensure its relevance, comprehensiveness, and comprehensibility for intended research.

Furthermore, ESM data can be compared to retrospective patient-reported outcome data to confirm and obtain more evidence on the added value of the ESM and the different experiences it captures and to investigate the ecological validity of such data. Another important area of future ESM research in oncology can be to explore its clinical value and utility, for instance, by providing clinicians with time-series visualizations of their patients and comparing these with information gathered through traditional consultations.

### Strengths and Limitations

This study is among the first studies to test the content validity of an ESM questionnaire in any scientific field and has resulted in the first content-valid ESM questionnaire in the field of oncology, thereby answering to recent calls for more questionnaire validation in ESM research [9,12,13]. This study has several strengths. First, it involved close collaboration with people with cancer and health care professionals in multiple phases of questionnaire development, ensuring its relevance for the target population. Second, relevance was further ensured by adapting items from existing validated PROMs [51-53]. Moreover, unlike many quantitatively focused questionnaires in ESM research, the use of a free-text response item “If there is anything else you want to mention about the period since last beep, you can do that here:” allows us to study any relevant experiences that are currently missing in the core questionnaire. Third, we included an equal number of patients aged <70 years and >70 years, ensuring the inclusion of the latter as an often underrepresented group in cancer studies. Finally, this study’s relatively good participation rate reduces the risk of selection bias.

Several limitations should be noted. First, the study was limited to Dutch-speaking patients from 2 study sites, possibly limiting the extent to which the ESM-AC questionnaire’s content validity can be generalized to patients with sociodemographic characteristics different from our sample. However, the ESM questionnaire will be further tested among new patients recruited from different hospitals. Second, the relatively high functional status of patients in our sample (ie, Eastern Cooperative Oncology Group scores between 0 and 2) may lead to limited generalizability of the results to patients with advanced cancer who have more functional limitations. Third, as no people aged >78 years participated, the usability of our ESM is unknown for older populations. Fourth, we did not record whether patients were actively receiving treatment, thereby preventing more detailed insight into the sample’s current perspectives and experiences. Finally, due to the study design, we were not able to test how health care professionals viewed the relevance and how patients and health care professionals viewed the relative importance of evening assessment items that that were initially removed by the authors based on their low expected within-day variability.

### Conclusions

We successfully developed the ESM-AC questionnaire, the first content-valid digital ESM questionnaire in oncology to study the daily experiences of people with advanced breast or lung cancer in their everyday environments. If the method proves feasible in future research on advanced cancer and in other patient groups, it paves the way toward gaining novel insights into the daily lives of patients with cancer, possibly informing and facilitating patient-centered care.

## Appendix

Multimedia Appendix 1 A figure about the criteria for categorization into the core questionnaire, supplementary set, or items to be removed.

Multimedia Appendix 2 Inappropriateness frequencies, relevance means, and proportions of relative importance ratings of experience sampling method items.

Multimedia Appendix 3 Frequency table of the categorized reasons for deeming an item inappropriate.

Multimedia Appendix 4 Content categories of patient and health care professional responses to the open-ended question on what content was missing from the item sets.

Multimedia Appendix 5 Proportions of participants who had no difficulties with comprehensibility of item per item, ordered by subdomain.

Multimedia Appendix 6 Resulting Dutch item versions before and after the first 2 interview rounds.

Multimedia Appendix 7 Final core the ESM-AC (Experience Sampling Method for People Living With Advanced Cancer) questionnaire.

Multimedia Appendix 8 Dutch onboarding session manual created after interview round 3.

## Abbreviations

COSMIN: Consensus-Based Standards for the Selection of Health Measurement Instruments
EORTC: European Organization for Research and Treatment of Cancer
EORTC QLQ-C30: European Organization for Research and Treatment of Cancer Core Quality of Life Questionnaire
ESM: experience sampling method
ESM-AC: Experience Sampling Method for People Living With Advanced Cancer
FACIT-Pal: Functional Assessment of Chronic Illness Therapy–Palliative Care
PROM: patient-reported outcome measure
