# Supplementary material for: Uncovering the Daily Experiences of People Living With Advanced Cancer Using an Experience Sampling Method Questionnaire: Development, Content Validation, and Optimization Study
Source: JMIR Cancer. 2024 Nov 5;10:e57510. doi: 10.2196/57510 (PMC11576598; doi:10.2196/57510)
Supplement: Multimedia Appendix 1 [file cancer_v10i1e57510_app1.docx]

**Supplementary material 1.** Figure on criteria for categorization into the core questionnaire, supplementary set or items to be removed.
