# Supplementary material for: Uncovering the Daily Experiences of People Living With Advanced Cancer Using an Experience Sampling Method Questionnaire: Development, Content Validation, and Optimization Study
Source: JMIR Cancer. 2024 Nov 5;10:e57510. doi: 10.2196/57510 (PMC11576598; doi:10.2196/57510)
Supplement: Multimedia Appendix 2 [file cancer_v10i1e57510_app2.docx]

**Multimedia Appendix 2.** Inappropriateness frequencies, relevance means, and proportions of relative importance ratings of experience sampling method items

| Subdomain | Item | Participants judging item as inappropriate (n) | | Relevance, mean (SD) | | | Participant who selected the item as one of the most important in the subdomain (n, %) | | |
| --- | --- | --- | --- | --- | --- | --- | --- | --- | --- |
|  |  | Patient | HCP^a^ | Patient | HCP | Mean (SD) of patient and HCP values | Patient | HCP | Patient and HCP values |
| **Initial items at round 1** |  |  |  |  |  |  |  |  |  |
| Global Well-Being | “At this moment, I am content with the quality of my life.” | 0 | 0 | 82.2 (27.8) | 79.2 (39.6) | 80.7  (2.2) | 13 (87) | 6 (75) | 19 (83) |
| Physical Functioning | “Did this pain interfere with your daily activities?” | 1 | 0 | 57.8 (40.8) | 91.7 (23.6) | 74.7  (24) | 9 (60) | 4 (50) | 13 (57) |
|  | “Since the last beep, I was limited in pursuing my hobbies or other leisure time activities.” | 1 | 0 | 76.2 (30.5) | 75 (34.5) | 75.6  (0.8) | 2 (13) | 2 (25) | 4 (17) |
|  | “Since the last beep, I had trouble meeting the needs of my family because of my physical condition.” | 1 | 0 | 64.4 (40.8) | 83.3 (25.2) | 73.9  (13.4) | 5 (33) | 2 (25) | 7 (30) |
|  | “Since the last beep, I needed help with eating, dressing, washing myself or using the toilet.” | 2 | 0 | 57.8 (38.8) | 95.8 (11.8) | 76.8  (26.9) | 4 (27) | 6 (75) | 10 (43) |
|  | “Since the last beep, I was affected by poor mobility.” | 1 | 0 | 71.1 (27.8) | 54.2 (43.4) | 62.6  (12) | 6 (40) | 2 (25) | 8 (35) |
|  | “Since the last beep, I had trouble doing strenuous activities, like carrying a heavy shopping bag or suitcase.” | 1 | 0 | 55.6 (32.5) | 58.3 (15.4) | 56.9  (2) | 4 (27) | 0 (0) | 4 (17) |
| Physical Symptoms | “At this moment, I am short of breath.” | 0 | 0 | 78.6 (21.1) | 87.5 (35.4) | 83  (6.3) | 5 (33) | 4 (50) | 9 (39) |
|  | “At this moment, I have the need to rest.” | 0 | 0 | 76.2 (20.4) | 54.2 (39.6) | 65.2  (15.6) | 4 (27) | 1 (12) | 5 (22) |
|  | “At this moment, my mouth and throat are dry.” | 0 | 0 | 37.8 (35.3) | 70.8 (27.8) | 54.3  (23.4) | 1 (7) | 0 (0) | 1 (4) |
|  | “At this moment, I lack appetite.” | 0 | 0 | 77.8 (20.6) | 91.7 (23.6) | 84.7  (9.8) | 5 (33) | 3 (38) | 8 (35) |
|  | “At this moment, I am bothered by side effects of treatment.” | 0 | 0 | 80 (24.6) | 70.8 (41.6) | 75.4  (6.5) | 9 (60) | 4 (50) | 13 (57) |
|  | “At this moment, I am constipated.” | 2 | 0 | 73.3 (25.8) | 87.5 (24.8) | 80.4  (10) | 4 (27) | 2 (25) | 6 (26) |
|  | “At this moment, I have pain.” | 1 | 0 | 77.8 (24.1) | 100 (0) | 88.9  (15.7) | 11 (73) | 7 (88) | 18 (78) |
|  | “At this moment, I have a lack of energy.” | 0 | 0 | 80 (21.1) | 83.3 (30.9) | 81.7  (2.4) | 6 (40) | 2 (25) | 8 (35) |
|  | “At this moment, I feel nauseated.” | 0 | 0 | 75.6 (23.5) | 87.5 (17.3) | 81.5  (8.4) | 5 (33) | 5 (62) | 10 (43) |
|  | “At this moment, I feel tired.” | 0 | 0 | 80 (21.1) | 100 (0) | 90  (14.1) | 6 (40) | 4 (50) | 10 (43) |
|  | “At this moment, I feel weak.” | 0 | 0 | 60 (31.4) | 41.7 (49.6) | 50.8  (13) | 1 (7) | 1 (12) | 2 (9) |
|  | “At this moment, I feel drowsy.” | 0 | 0 | 51.1 (27.8) | 37.5 (41.6) | 44.3  (9.6) | 1 (7) | 0 (0) | 1 (4) |
|  | “At this moment, I feel ill.” | 0 | 0 | 69 (35.7) | 37.5 (37.5) | 53.3  (22.3) | 4 (27) | 2 (25) | 6 (26) |
|  | “At this moment, I have swelling in parts of my body.” | 0 | 1 | 59 (27.7) | 58.3 (29.6) | 58.6  (0.5) | 1 (7) | 1 (12) | 2 (9) |
|  | “Since the last beep, I have had diarrhea.” | 2 | 0 | 66.7 (30.9) | 83.3 (25.2) | 75  (11.8) | 3 (20) | 1 (12) | 4 (17) |
|  | “Since the last beep, I have had to vomit.” | 0 | 0 | 68.9 (34.4) | 58.3 (38.8) | 63.6  (7.5) | 1 (7) | 2 (25) | 3 (13) |
| Negative Affect | “At this moment, I feel anxious.” | 0 | 0 | 76.2 (33.2) | 100 (0) | 88.1  (16.8) | 8 (53) | 7 (88) | 15 (65) |
|  | “At this moment, I feel lonely.” | 0 | 0 | 78.6 (24.7) | 91.7 (15.4) | 85.1  (9.3) | 4 (27) | 4 (50) | 8 (35) |
|  | “At this moment, I feel irritated.” | 0 | 0 | 40 (28.7) | 54.2 (39.6) | 47.1  (10) | 1 (7) | 0 (0) | 1 (4) |
|  | “At this moment, I feel tense.” | 0 | 0 | 48.9 (35.3) | 45.8 (43.4) | 47.4  (2.2) | 1 (7) | 1 (12) | 2 (9) |
|  | “At this moment, I feel stressed.” | 0 | 0 | 57.8 (32) | 58.3 (42.7) | 58.1  (0.4) | 3 (20) | 0 (0) | 3 (13) |
|  | “At this moment, I feel listless.” | 0 | 0 | 46.7 (35.2) | 75 (29.6) | 60.8  (20) | 3 (20) | 0 (0) | 3 (13) |
|  | “At this moment, I feel depressed.“ | 0 | 0 | 62.2 (27.8) | 66.7 (43.6) | 64.4  (3.1) | 1 (7) | 1 (12) | 2 (9) |
|  | “At this moment, I feel nervous.” | 0 | 0 | 53.3 (30.3) | 58.3 (42.7) | 55.8  (3.5) | 2 (13) | 1 (12) | 3 (13) |
|  | “At this moment, I feel irritable.” | 0 | 0 | 51.1 (30.5) | 70.8 (21.4) | 61  (13.9) | 2 (13) | 0 (0) | 2 (9) |
|  | “At this moment, I feel down.” | 0 | 0 | 60 (33.8) | 54.2 (46.9) | 57.1  (4.1) | 1 (7) | 0 (0) | 1 (4) |
|  | “At this moment, I feel sad.” | 0 | 0 | 68.9 (23.5) | 91.7 (15.4) | 80.3  (16.1) | 2 (13) | 2 (25) | 4 (17) |
| Positive Affect | “At this moment, I feel energetic.” | 2 | 0 | 57.1 (33.2) | 70.8 (27.8) | 64  (9.7) | 6 (40) | 4 (50) | 10 (43) |
|  | “At this moment, I feel enthusiastic.” | 4 | 1 | 40.5 (32.5) | 54.2 (43.4) | 47.3  (9.7) | 3 (20) | 0 (0) | 3 (13) |
|  | “At this moment, I feel calm.” | 1 | 0 | 40.5 (29.8) | 70.8 (27.8) | 55.7  (21.5) | 4 (27) | 3 (38) | 7 (30) |
|  | “At this moment, I feel relaxed.” | 2 | 0 | 61.9 (34.2) | 79.2 (30.5) | 70.5  (12.2) | 4 (27) | 3 (38) | 7 (30) |
|  | “At this moment, I feel cheerful.” | 2 | 3 | 60 (31.4) | 62.5 (37.5) | 61.2  (1.8) | 2 (13) | 2 (25) | 4 (17) |
|  | “At this moment, I feel satisfied.” | 2 | 0 | 69 (20.5) | 79.2 (35.4) | 74.1  (7.2) | 7 (47) | 4 (50) | 11 (48) |
| Cognitive Complaints | “At this moment, my thinking is clear.” | 0 | 0 | 57.1 (33.2) | 75 (38.8) | 66.1  (12.6) | 3 (20) | 1 (12) | 4 (17) |
|  | “Since the last beep, I have had difficulty in concentrating on things, like reading a newspaper or watching television.” | 0 | 0 | 66.7 (32) | 100 (0) | 83.3  (23.6) | 7 (47) | 6 (75) | 13 (57) |
|  | “Since the last beep, I have had difficulty remembering things.” | 0 | 0 | 64.4 (32) | 95.8 (11.8) | 80.1  (22.2) | 5 (33) | 1 (12) | 6 (26) |
| Psychological Well-being | “At this moment, I am satisfied with how I am coping with my illness.” | 0 | 1 | 86.7 (16.9) | 70.8 (41.6) | 78.7  (11.2) | 13 (87) | 6 (75) | 19 (83) |
|  | “At this moment, I have peace of mind.” | 0 | 0 | 73.3 (28.7) | 58.3 (42.7) | 65.8  (10.6) | 5 (33) | 5 (62) | 10 (43) |
|  | “At this moment, I am able to enjoy life.” | 2 | 0 | 77.8 (30) | 70.8 (33) | 74.3  (4.9) | 12 (80) | 4 (50) | 16 (70) |
|  | “At this moment, I worry.” | 0 | 0 | 75.6 (29.5) | 87.5 (17.3) | 81.5  (8.4) | 12 (80) | 8 (100) | 20 (87) |
| Sleep Quality | “Last night, I slept well.” | 0 | 0 | 86.7 (21.1) | 95.8 (11.8) | 91.2  (6.5) | 14 (93) | 8 (100) | 22 (96) |
| Social Well-being | “At this moment, I feel like a burden to my family.” | 1 | 1 | 73.8 (26.7) | 87.5 (35.4) | 80.7  (9.7) | 13 (87) | 7 (88) | 20 (87) |
|  | “At this moment, I get emotional support from the people close to me.” | 1 | 0 | 95.2 (12.1) | 91.7 (23.6) | 93.5  (2.5) | 15 (100) | 7 (88) | 22 (96) |
| Spiritual and Existential Well-being | “At this moment, I accept my illness.” | 0 | 0 | 88.9 (27.2) | 95.8 (11.8) | 92.4  (4.9) | 13 (87) | 3 (38) | 16 (70) |
|  | “At this moment, I am able to make decisions.” | 2 | 0 | 44.4 (27.2) | 41.7 (42.7) | 43.1  (2) | 1 (7) | 1 (12) | 2 (9) |
|  | “At this moment, I feel hopeful.” | 0 | 0 | 73.3 (31.4) | 95.8 (11.8) | 84.6  (15.9) | 8 (53) | 4 (50) | 12 (52) |
|  | “At this moment, I feel useful.” | 2 | 0 | 54.8 (33.6) | 83.3 (25.2) | 69  (20.2) | 4 (27) | 4 (50) | 8 (35) |
|  | “At this moment, I feel independent.” | 2 | 0 | 71.1 (30.5) | 70.8 (45.2) | 71  (0.2) | 4 (27) | 4 (50) | 8 (35) |
| **Items added after round 1** |  |  |  |  |  |  |  |  |  |
| Physical Functioning | “Today, I did everything that I wanted to do.” | 0 | 0 | 64.6 (31) | -^b^ | - | - | - | - |
| Physical Symptoms | “The neuropathy I experience is located at:” | 0 | 0 | 77.1 (35.9) | - | - | - | - | - |
|  | “At this moment, I am experiencing neuropathy (e.g., tingling or pain).” | 0 | 0 | 81.3 (34.4) | - | - | - | - | - |
| Negative Affect | “At this moment, I feel angry.” | 0 | 0 | 56.3 (33.8) | - | - | - | - | - |
| Positive Affect | “At this moment, I feel cheerful.” | 0 | 0 | 54.2 (40.1) | - | - | - | - | - |
| Professional Life | “At this moment, I feel capable of working.” | 0 | 0 | 85.7 (21.5) | - | - | - | - | - |
| Psychological Well-being | “At this moment, I have negative thoughts or feelings.” | 0 | 0 | 82.2 (21.3) | - | - | - | - | - |
|  | “Today, my work (including household chores) has given me satisfaction.” | 0 | 0 | 62.5 (26.9) | - | - | - | - | - |
| Sleep Quality | “Last night, I woke up ... times.“ [0 - 1-2 - 3-4 - 5-6 - More than 6] | 0 | 0 | 54.2 (40.1) | - | - | - | - | - |
|  | “Last night, I had trouble falling back asleep after waking up.” | 0 | 0 | 70.8 (31.9) | - | - | - | - | - |
| Social Well-being | “I am satisfied with the level of intimacy I experienced yesterday.” | 0 | 0 | 68.8 (28.5) | - | - | - | - | - |
|  | “Today, I felt that my family appreciates me.” | 0 | 0 | 64.6 (37.5) | - | - | - | - | - |
|  | “Today, I worried about my loved ones.” | 0 | 0 | 68.8 (25.7) | - | - | - | - | - |
|  | “Today, I was able to openly discuss my concerns with my loved ones.” | 0 | 0 | 68.8 (31) | - | - | - | - | - |
|  | “Today, someone in my family or friends felt anxious or concerned about me.” | 0 | 0 | 60.4 (40.8) | - | - | - | - | - |
|  | “Today, my physical condition or treatment disrupted my social activities.” | 0 | 0 | 83.3 (24.4) | - | - | - | - | - |
|  | “Today, my physical condition or treatment disrupted my family life.” | 0 | 0 | 71.1 (37.5) | - | - | - | - | - |
|  | “Today, I was satisfied with the communication about my illness with my loved ones.” | 0 | 0 | 62.5 (34.2) | - | - | - | - | - |
| Spiritual and Existential Well-being | “Today, I felt connected to my faith.” | 0 | 0 | 35.7 (38) | - | - | - | - | - |
|  | “Today, I made the most of my day.” | 0 | 0 | 72.9 (34.9) | - | - | - | - | - |
|  | “Today, I found life meaningful.” | 0 | 0 | 72.9 (32.7) | - | - | - | - | - |

^a^HCP = Health care professional

^b^Not measured.
