# Supplementary material for: Uncovering the Daily Experiences of People Living With Advanced Cancer Using an Experience Sampling Method Questionnaire: Development, Content Validation, and Optimization Study
Source: JMIR Cancer. 2024 Nov 5;10:e57510. doi: 10.2196/57510 (PMC11576598; doi:10.2196/57510)
Supplement: Multimedia Appendix 3 [file cancer_v10i1e57510_app3.docx]

**Multimedia Appendix 3.** Frequency table of categorized reasons for deeming item inappropriate.

| **Theme** | **Item** | **poor clinical utility** | **confronting** | **Content not fitting under domain** | **Overlap with other items** | **privacy** | **question formulation** | **Little within-day variability** | **Not specified** |
| --- | --- | --- | --- | --- | --- | --- | --- | --- | --- |
| Global Well-Being | At this moment, I am content with the quality of my life. | 0 | 0 | 0 | 0 | 0 | 0 | 0 | 0 |
| Physical Functioning | Did this pain interfere with your daily activities? | 0 | 0 | 0 | 1 | 0 | 0 | 0 | 0 |
|  | Since the last beep, I was limited in pursuing my hobbies or other leisure time activities. | 0 | 0 | 0 | 1 | 0 | 0 | 0 | 0 |
|  | Since the last beep, I had trouble meeting the needs of my family because of my physical condition. | 0 | 0 | 0 | 1 | 0 | 0 | 0 | 0 |
|  | Since the last beep, I needed help with eating, dressing, washing myself or using the toilet. | 0 | 0 | 0 | 1 | 1 | 0 | 0 | 0 |
|  | Since the last beep, I was affected by poor mobility. | 0 | 0 | 0 | 1 | 0 | 0 | 0 | 0 |
|  | Since the last beep, I had trouble doing strenuous activities, like carrying a heavy shopping bag or suitcase. | 0 | 0 | 0 | 1 | 0 | 0 | 0 | 0 |
| Physical Symptoms | At this moment, I am short of breath. | 0 | 0 | 0 | 0 | 0 | 0 | 0 | 0 |
|  | At this moment, I have the need to rest. | 0 | 0 | 0 | 0 | 0 | 0 | 0 | 0 |
|  | At this moment, my mouth and throat are dry. | 0 | 0 | 0 | 0 | 0 | 0 | 0 | 0 |
|  | At this moment, I lack appetite. | 0 | 0 | 0 | 0 | 0 | 0 | 0 | 0 |
|  | At this moment, I am bothered by side effects of treatment. | 0 | 0 | 0 | 0 | 0 | 0 | 0 | 0 |
|  | At this moment, I am constipated. | 0 | 0 | 0 | 0 | 2 | 0 | 0 | 0 |
|  | At this moment, I have pain. | 0 | 0 | 0 | 0 | 0 | 1 | 0 | 0 |
|  | At this moment, I have a lack of energy. | 0 | 0 | 0 | 0 | 0 | 0 | 0 | 0 |
|  | At this moment, I feel nauseated. | 0 | 0 | 0 | 0 | 0 | 0 | 0 | 0 |
|  | At this moment, I feel tired. | 0 | 0 | 0 | 0 | 0 | 0 | 0 | 0 |
|  | At this moment, I feel weak. | 0 | 0 | 0 | 0 | 0 | 0 | 0 | 0 |
|  | At this moment, I feel drowsy. | 0 | 0 | 0 | 0 | 0 | 0 | 0 | 0 |
|  | At this moment, I feel ill. | 0 | 0 | 0 | 0 | 0 | 0 | 0 | 0 |
|  | At this moment, I have swelling in parts of my body. | 0 | 0 | 0 | 0 | 0 | 1 | 0 | 0 |
|  | Since the last beep, I have had diarrhea. | 0 | 0 | 0 | 0 | 2 | 0 | 0 | 0 |
|  | Since the last beep, I have had to vomit. | 0 | 0 | 0 | 0 | 0 | 0 | 0 | 0 |
| Negative Affect | At this moment, I feel anxious. | 0 | 0 | 0 | 0 | 0 | 0 | 0 | 0 |
|  | At this moment, I feel lonely. | 0 | 0 | 0 | 0 | 0 | 0 | 0 | 0 |
|  | At this moment, I feel irritated. | 0 | 0 | 0 | 0 | 0 | 0 | 0 | 0 |
|  | At this moment, I feel tense. | 0 | 0 | 0 | 0 | 0 | 0 | 0 | 0 |
|  | At this moment, I feel stressed. | 0 | 0 | 0 | 0 | 0 | 0 | 0 | 0 |
|  | At this moment, I feel listless. | 0 | 0 | 0 | 0 | 0 | 0 | 0 | 0 |
|  | At this moment, I feel depressed. (translation EORTC QLQ-C30 EN-NL) | 0 | 0 | 0 | 0 | 0 | 0 | 0 | 0 |
|  | At this moment, I feel nervous. | 0 | 0 | 0 | 0 | 0 | 0 | 0 | 0 |
|  | At this moment, I feel irritable. | 0 | 0 | 0 | 0 | 0 | 0 | 0 | 0 |
|  | At this moment, I feel down. | 0 | 0 | 0 | 0 | 0 | 0 | 0 | 0 |
|  | At this moment, I feel sad. | 0 | 0 | 0 | 0 | 0 | 0 | 0 | 0 |
| Positive Affect | At this moment, I feel energetic. | 0 | 1 | 0 | 1 | 0 | 0 | 0 | 0 |
|  | At this moment, I feel enthusiastic. | 0 | 0 | 0 | 2 | 0 | 0 | 2 | 1 |
|  | At this moment, I feel calm. | 0 | 0 | 0 | 1 | 0 | 0 | 0 | 0 |
|  | At this moment, I feel relaxed. | 0 | 0 | 0 | 2 | 0 | 0 | 0 | 0 |
|  | At this moment, I feel cheerful. | 0 | 2 | 0 | 1 | 0 | 0 | 1 | 1 |
|  | At this moment, I feel satisfied. | 0 | 0 | 0 | 1 | 0 | 0 | 1 | 0 |
| Cognitive Complaints | At this moment, my thinking is clear. | 0 | 0 | 0 | 0 | 0 | 0 | 0 | 0 |
|  | Since the last beep, I have had difficulty in concentrating on things, like reading a newspaper or watching television. | 0 | 0 | 0 | 0 | 0 | 0 | 0 | 0 |
|  | Since the last beep, I have had difficulty remembering things. | 0 | 0 | 0 | 0 | 0 | 0 | 0 | 0 |
| Psychological Well-Being | At this moment, I am satisfied with how I am coping with my illness. | 0 | 0 | 0 | 0 | 0 | 1 | 0 | 0 |
|  | At this moment, I have peace of mind. | 0 | 0 | 0 | 0 | 0 | 0 | 0 | 0 |
|  | At this moment, I am able to enjoy life. | 0 | 1 | 0 | 0 | 0 | 0 | 1 | 0 |
|  | At this moment, I worry. | 0 | 0 | 0 | 0 | 0 | 0 | 0 | 0 |
| Sleep Quality | Last night, I slept well. | 0 | 0 | 0 | 0 | 0 | 0 | 0 | 0 |
| Social Well-Being | At this moment, I feel like a burden to my family. | 0 | 1 | 0 | 0 | 0 | 0 | 0 | 1 |
|  | At this moment, I get emotional support from the people close to me. | 0 | 1 | 0 | 0 | 0 | 0 | 0 | 0 |
| Spiritual & Existential Well-Being | At this moment, I accept my illness. | 0 | 0 | 0 | 0 | 0 | 0 | 0 | 0 |
|  | At this moment, I am able to make decisions. | 1 | 0 | 0 | 0 | 0 | 1 | 0 | 0 |
|  | At this moment, I feel hopeful. | 0 | 0 | 0 | 0 | 0 | 0 | 0 | 0 |
|  | At this moment, I feel useful. | 0 | 2 | 0 | 0 | 0 | 0 | 0 | 0 |
|  | At this moment I feel independent. | 0 | 0 | 1 | 0 | 0 | 0 | 1 | 0 |
