# Supplementary material for: Uncovering the Daily Experiences of People Living With Advanced Cancer Using an Experience Sampling Method Questionnaire: Development, Content Validation, and Optimization Study
Source: JMIR Cancer. 2024 Nov 5;10:e57510. doi: 10.2196/57510 (PMC11576598; doi:10.2196/57510)
Supplement: Multimedia Appendix 4 [file cancer_v10i1e57510_app4.docx]

**Multimedia Appendix 4.** Content categories of patient and healthcare professional responses to the open-ended question on what content was missing from the item sets

| **Domain** | **Category** | **Patients** | **Healthcare professionals** |
| --- | --- | --- | --- |
| Global Well-Being | reason for low experienced quality of life | 0 | 1 |
|  | finding life worthwhile | 0 | 1 |
| Physical Functioning | additional example for strenuous activities: using stairs | 1 | 0 |
|  | being able to walk with others | 1 | 0 |
|  | reasons why participant has bad physical functioning | 0 | 1 |
|  | being able to go outside (for example, to take a walk) | 0 | 1 |
|  | how much time spent in bed or on couch | 0 | 1 |
|  | lagging behind with organisational aspects (example: household) | 0 | 1 |
| Physical Symptoms | sores and clefts in mouth | 1 | 0 |
|  | problems with easy opening wound | 1 | 0 |
|  | neuropathy: unpleasant sensation or tingling toes and fingers | 2 | 0 |
|  | unpleasant sensation around surgery site | 1 | 0 |
|  | split nails | 1 | 0 |
|  | runny nose | 1 | 0 |
|  | accelerated aging process and wear: eyes, ears, bones | 1 | 0 |
|  | trouble swallowing | 1 | 0 |
|  | nose bleeding | 1 | 0 |
|  | muscle acidification | 1 | 0 |
|  | stuffy nose | 1 | 0 |
|  | being bothered by tingling toes or fingers | 1 | 0 |
|  | coughing and coughing fits | 1 | 0 |
|  | dry skin | 1 | 0 |
|  | response option to mention additional experienced symptoms | 2 | 1 |
|  | tinnitus | 1 | 0 |
|  | neuropathy with location | 3 | 0 |
|  | neuropathic pain | 2 | 2 |
|  | cramps | 1 | 0 |
|  | stiffness in hands | 1 | 0 |
|  | vision problems | 0 | 1 |
|  | strength in arms and legs | 0 | 1 |
|  | incontinence | 0 | 1 |
|  | fever | 0 | 1 |
|  | pain location: head | 0 | 2 |
|  | pain location: back | 1 | 0 |
|  | pain location: bones | 1 | 0 |
|  | pain location: joints | 0 | 1 |
| Negative Affect | anger | 1 | 1 |
|  | target of anger (family, friends, physicians, nursing staff, everyone) |  | 1 |
|  | feelings barometer | 1 | 1 |
| Positive Affect | gratitude | 0 | 1 |
| Cognitive Complaints | reasons for low concentration levels | 2 |  |
|  | trouble multitasking | 0 | 1 |
|  | being able to formulate one’s opinion | 0 | 1 |
|  | additional example for concentration: being able to follow conversations | 0 | 1 |
|  | experiencing difficulties with processing received healthcare information | 0 | 1 |
|  | distinguishing between short and long-term memory | 0 | 1 |
|  | activity involving concentration problems | 0 | 1 |
|  | unable to think about other things (constant worry, rumination) | 0 | 1 |
| Psychological Well-Being | feeling misunderstood about symptoms (example: persistent fatigue) | 1 | 0 |
|  | experiencing dark thoughts or feelings | 0 | 2 |
|  | having something to look forward to | 1 | 0 |
|  | feeling able to pick up normal life | 1 | 0 |
|  | object of worry | 1 | 0 |
|  | handing over role within household | 0 | 1 |
|  | feeling guilty about handing over role within household | 0 | 1 |
|  | fear of permanent neuropathic pain | 0 | 1 |
|  | extent to which people can go for a good chat about their own problems | 0 | 2 |
|  | trust in partner | 0 | 1 |
|  | trust in healthcare | 1 | 2 |
|  | extent to which experience psychological support need | 0 | 1 |
|  | capacity to continue life in the current way | 0 | 2 |
|  | thoughts of the last hour | 0 | 1 |
|  | satisfaction with appearance (after mastectomy or hair loss) | 1 | 0 |
|  | worry about close others | 0 | 2 |
|  | feeling optimistic versus feeling pessimistic | 0 | 1 |
| Sleep Quality | frequency of waking up at night | 3 | 1 |
|  | being able to fall asleep after waking up at night | 2 | 0 |
|  | use of sleep medication | 1 | 0 |
|  | reason for waking up at night: toilet | 1 | 0 |
|  | reason for waking up at night: leg cramps | 1 | 0 |
|  | reason(s) for poor sleep | 2 | 5 |
|  | time spent resting during the day | 0 | 1 |
|  | lying awake | 0 | 1 |
|  | feeling rested | 0 | 1 |
| Social Well-Being | finding it difficult to talk to strangers about illness | 1 | 0 |
|  | quality of and opportunities for social life relative to the past (example: being able to go to bar) | 1 | 0 |
|  | extent to which unsolicited compassion is experienced | 1 | 0 |
|  | reason why feeling like a burden | 1 | 0 |
|  | disappointment in friends | 0 | 1 |
|  | distinguishing between family and friends | 0 | 2 |
|  | social isolation | 0 | 1 |
|  | feeling connected with others | 0 | 3 |
|  | open question for more info on social situation | 0 | 1 |
|  | outline family situation at start of study | 0 | 1 |
|  | extent to which you feel sufficiently supported by: relatives, care providers | 0 | 1 |
|  | who helped out today | 0 | 1 |
|  | extent to which able to participate in social events | 0 | 1 |
|  | talking to kids about one’s cancer | 1 | 0 |
|  | preventative withholding from activities with close others | 1 | 0 |
| Spiritual & Existential Well-Being | how close people are to their faith | 2 | 1 |
|  | greater need for or interest in spirituality | 1 | 0 |
|  | reason for feeling useful | 1 | 0 |
|  | reason for feeling dependent | 1 | 0 |
|  | extent to which occupied with death (example: arranging ones own funeral) | 1 | 0 |
|  | attitude towards death (open question) | 0 | 2 |
|  | level of anxiety towards end of life | 0 | 1 |
|  | prayed or lit a candle | 0 | 1 |
|  | finding life meaningful | 0 | 2 |
|  | able to find strength in difficult times | 0 | 1 |
|  | reflection on the meaningfulness of current day in one’s life | 0 | 1 |
|  | until when does the patient make plans (time perspective) | 0 | 1 |
|  | acceptance of limited time perspective | 0 | 1 |
|  | afraid of the future | 0 | 1 |
| Professional Life | quality of professional life | 1 | 0 |
|  | feeling capable to work | 1 | 1 |
| Sexual Well-Being | degree to which sexually active and how this impacts the patient | 2 | 2 |
|  | impact of sexuality on partner | 1 | 1 |
|  | impact of sexuality on relationship with partner | 1 | 1 |
| Care | frequency of medical contacts | 0 | 1 |
|  | unresolved issues and the capability of the patient to resolve those issues | 0 | 1 |
|  | experiencing difficulties with medication (scheme, a lot at once, knowing when, fear of becoming dependent) | 0 | 1 |
|  | received sufficient information | 0 | 1 |
|  | quality of care | 1 | 0 |
| Other | open question for additional experiences or explanations | 0 | 1 |
