# Supplementary material for: Uncovering the Daily Experiences of People Living With Advanced Cancer Using an Experience Sampling Method Questionnaire: Development, Content Validation, and Optimization Study
Source: JMIR Cancer. 2024 Nov 5;10:e57510. doi: 10.2196/57510 (PMC11576598; doi:10.2196/57510)
Supplement: Multimedia Appendix 5 [file cancer_v10i1e57510_app5.docx]

**Multimedia Appendix 5.** Proportions of participants that had no difficulties with comprehensibility of item per item, ordered by subdomain

| **Questionnaire** | **Subdomain** | **Item** | **Proportion of participants that had no difficulties with comprehensibility of item** |
| --- | --- | --- | --- |
| Core | Physical symptoms | At this moment, I have pain. | 0.83 |
|  |  | *If pain > 10:* The pain is located at the following body parts: [indicate the location(s) of pain on the doll]. | 0.83 |
|  |  | At this moment, I feel tired. | 1 |
|  |  | At this moment, I feel nauseated. | 1 |
|  |  | At this moment, I’m experiencing breathing problems (shortness of breath, difficulty breathing). | 1 |
|  | Negative affect | At this moment, I feel restless. | 1 |
|  |  | At this moment, I feel sad. | 1 |
|  | Positive affect | At this moment, I feel content. | 0.94 |
|  |  | At this moment, I feel relaxed. | 1 |
|  |  | At this moment, I feel energized. | 1 |
|  | Cognitive complaints | Since last beep, I had trouble concentrating on things like reading a newspaper, watching television or following a conversation. | 1 |
|  | Psychological well-being | At this moment, I feel worried. | 1 |
|  |  | At this moment, I feel depressed. | 1 |
|  |  | At this moment, I feel anxious. | 1 |
|  | Social well-being | At this moment, I feel lonely. | 0.89 |
|  | Global well-being | At this moment, I feel … [very bad – very good] | 0.94 |
|  |  | If there is anything else you want to note about the period since last beep, you can do it here: | 1 |
| Core: morning | Sleep quality | This night, I slept well. | 0.89 |
|  |  | *If sleep > 10:* I think I slept less well, because: | 1 |
| Core: evening | Physical functioning | Today, due to my physical condition, I had difficulty performing my daily activities. | 0.72 |
|  | Psychological well-being | I feel like I was able to enjoy my day today. | 0.89 |
|  | Social well-being | Today I received the support I needed from my loved one(s). | 0.83 |
|  |  | Today I felt like I was a burden to my loved one(s). | 0.94 |
|  | Spiritual-Existential well-being | Today I felt hopeful. | 0.94 |
| Supplementary | Physical symptoms | At this moment, I have a dry mouth or throat. | 1 |
|  |  | At this moment, I have a need to rest. | 1 |
|  |  | At this moment, I feel weak. | 1 |
|  |  | At this moment, I suffer from nerve damage (e.g. tingling or pain). | 0.94 |
|  |  | *If neuropathy > 10:* The nerve damage I experienced was located at: | 1 |
|  |  | At this moment, I have constipation. | 1 |
|  |  | Since last beep, I have vomited. | 1 |
|  |  | Since last beep, I have had diarrhea. | 1 |
|  | Negative affect | At this moment, I feel nervous. | 1 |
|  |  | At this moment, I feel tense. | 1 |
|  |  | At this moment, I feel stressed. | 1 |
|  |  | At this moment, I feel irritable. | 1 |
|  |  | At this moment, I feel down. | 1 |
|  |  | At this moment, I feel lethargic. | 0.94 |
|  |  | At this moment, I feel angry. | 1 |
|  | Positive affect | At this moment, I feel happy. | 1 |
|  |  | At this moment, I feel calm. | 0.94 |
|  | Cognitive complaints | Since last beep, I had trouble remembering things. | 1 |
|  | Psychological well-being | At this moment, I have negative thoughts or feelings. | 1 |
|  | Professional well-being | At this moment, I feel capable of working | 1 |
| Supplementary: morning | Sleep quality | Last night, I woke up … times. [0 - 1-2 - 3-4 - 5-6 – More than 6] | 1 |
|  |  | Last night, I had trouble getting back to sleep after getting up. | 1 |
|  | Social well-being | I am satisfied with the extent to which I experienced intimacy with my partner yesterday. | 0.78 |
| Supplementary: evening | Physical well-being | Today I had no appetite. | 0.94 |
|  | Physical well-being | Today some parts of my body were swollen. | 1 |
|  | Physical functioning | I’m satisfied with all I was able to do today. | 0.78 |
|  | Psychological well-being | Today my work (including household) has given me satisfaction. | 0.94 |
|  | Social well-being | Today I have been worried about my loved ones. | 1 |
|  |  | Today my physical condition or treatment has interfered with my family life. | 0.72 |
|  |  | Today my physical condition or treatment has interfered with my social activities. | 0.83 |
|  |  | Today I was able to openly discuss my concerns with my loved ones. | 0.89 |
|  |  | Today I was satisfied with the communication about my illness with my loved ones. | 0.78 |
|  |  | Today I felt that my family appreciates me. | 1 |
|  |  | Today, one of my family or friends has felt anxious or worried about me. | 0.94 |
|  | Spiritual-existential well-being | Today I felt useful. | 1 |
|  |  | Today I felt connected to my faith. | 1 |
|  |  | Today I found life meaningful. | 1 |
|  |  | Today I got as much out of my day as possible. | 1 |
| Core | Social company | With whom am I? [Partner - Child(ren) – Other family members – Friend(s) – Acquaintance(s) - Nobody – Healthcare provider – Co-worker(s) - Online contact – Cleaning help - Others] | 0.78 |
|  |  | *If not ‘Nobody (I am alone)’:* I think this company is pleasant. | 1 |
|  |  | *If ‘Nobody (I am alone)’:* It feels okay to be alone. | 1 |
|  | Activity | What am I doing? (right before the beep went off) [Active leisure (walking, cycling, odd jobs, …) – Passive leisure (watching tv, internet, something quiet, …) - Work - Households, groceries, home administration - En route - Self-care, personal hygiene (washing, dressing, …) - Eating, drinking - Taking care of my (grand)child - Conversation, interaction – Sleeping - Resting - Nothing – Something else] | 0.89 |
|  |  | *If not 'Nothing':* I liked this activity. | 0.94 |
|  |  | *If not ‘Nothing’:* I felt limited doing this activity. | 0.83 |
|  |  | *If not ‘Nothing’:* Right now, this activity is difficult for me. | 0.94 |
|  | Medication | Since last beep, I have used the following: [Medication - Cigarettes - Alcohol - Caffeine - Nothing – Other substances] | 1 |
|  |  | *If ‘Medication’:* I used medication against: [Pain - Nausea - Others: …] | 1 |
|  | Location | Where am I? [At home – At someone else’s home - Store - Hospital - Work] | 1 |
|  |  | *If ‘At home’, ‘At someone else’s home’, or ‘Hospital’:* I’m in bed or on the couch. | 1 |
|  |  | I would rather be somewhere else. | 0.83 |
|  | Events | Think of the most pleasant event or activity since the last beep. How pleasant was it? | 0.89 |
|  |  | The pleasant event was related to… | 1 |
|  |  | Think of the most unpleasant event or activity since the last beep. How unpleasant was it? | 0.72 |
|  |  | The unpleasant event was related to… | 1 |
