# Supplementary material for: Uncovering the Daily Experiences of People Living With Advanced Cancer Using an Experience Sampling Method Questionnaire: Development, Content Validation, and Optimization Study
Source: JMIR Cancer. 2024 Nov 5;10:e57510. doi: 10.2196/57510 (PMC11576598; doi:10.2196/57510)
Supplement: Multimedia Appendix 6 [file cancer_v10i1e57510_app6.docx]

**Multimedia Appendix 6.** Resulting Dutch item versions before and after the first two interview rounds.

| **Domain before Round 1** | **Initial Dutch item source** | **In-the-moment version** | **Domain after Round 1** | **Round 1 Results** | **Category after Round 1** | **Domain after Round 2** | **Round 2 Results** | **Category after Round 2** |
| --- | --- | --- | --- | --- | --- | --- | --- | --- |
| Algemeen welzijn | Facit-Pal | Op dit moment ben ik tevreden met de kwaliteit van mijn leven. | Algemeen welzijn | Op dit moment voel ik me | core | Algemeen welzijn | Op dit moment voel ik me … | core |
|  | PETRA |  | Algemeen welzijn | Als u nog iets over de periode sinds de vorige biep wilt noteren, dan kunt u dat hier doen: | core | Algemeen welzijn | Als u nog iets over de periode sinds de vorige biep wilt noteren, dan kunt u dat hier doen: | core |
|  | ESM Item Repository |  | Context: Aanwezige mensen | Met wie ben ik? ¨ Partner ¨ Kind(eren) ¨ Andere familieleden ¨ Vriend(en) ¨ Kennis(sen) ¨ Onbekende mensen ¨ Niemand – Ik ben alleen ¨ Zorgverlener ¨ Collega(‘s) ¨ Online contact ¨ Poetshulp ¨ Anderen: _____________________ | core | Context: Aanwezige mensen | Met wie ben ik? o Partner o Kind(eren) o Andere familieleden o Vriend(en) o Kennis(sen) o Zorgverlener o Collega(‘s) o Online contact (bv. Whatsapp) of telefoneren o Anderen o Niemand (ik ben alleen) | core |
|  | ESM Item Repository |  | Context: Aanwezige mensen | (Als bij anderen) Ik vind dit gezelschap aangenaam. | core | Context: Aanwezige mensen | *Als niet alleen:* Ik vind dit gezelschap aangenaam. | core |
|  | ESM Item Repository |  | Context: Aanwezige mensen | (Als alleen) Het voelt oké om alleen te zijn. | core | Context: Aanwezige mensen | *Als alleen:* Het voelt oké om alleen te zijn. | core |
|  | ESM Item Repository |  | Context: Activiteiten | Wat doe ik? (vlak voor de biep afging) ¨ Actieve vrije tijdsbesteding (wandelen, fietsen, klussen, …) ¨ Passieve vrije tijdsbesteding (tv kijken, internetten, iets rustig, …) ¨ Werk of taken voor school/universiteit ¨ Huishouden, boodschappen, thuisadministratie ¨ Onderweg ¨ Zelfverzorging, persoonlijke hygiëne (wassen, aankleden, …) ¨ Eten, drinken ¨ Zorgen voor (klein)kinderen ¨ Gesprek, interactie ¨ Slapen ¨ Rusten ¨ Niets ¨ Iets anders: __________________________ | core | Context: Activiteiten | Wat deed ik vlak voor de biep afging? o Actieve vrije tijdsbesteding (wandelen, fietsen, klussen, …) o Passieve vrije tijdsbesteding (tv kijken, internetten, iets rustig, …) o Werk o Huishouden, boodschappen, thuisadministratie o Onderweg (bv. op de bus) o Zelfverzorging, persoonlijke hygiëne o Eten, drinken o Zorgen voor (klein)kinderen o Gesprek, interactie o Slapen o Niets o Iets anders | core |
|  | ESM Item Repository |  | Context: Activiteiten | Ik vind deze activiteit leuk. | core | Context: Activiteiten | Ik vond de activiteit die ik vlak voor de biep deed leuk. | core |
|  | EORTC QLQ-C30 |  | Context: Activiteiten | Ik voel me beperkt bij het doen van deze activiteit. | core | Context: Activiteiten | Ik voelde me beperkt bij het doen van de activiteit die ik vlak voor de biep deed. | core |
|  | ESM Item Repository |  | Context: Locatie | Waar ben ik? ¨ Thuis ¨ Bij iemand anders thuis ¨ Winkel ¨ Ziekenhuis ¨ Werk ¨ Ergens anders: __________________ | core | Context: Locatie | Waar ben ik?  o Thuis o Bij iemand anders thuis o Winkel o Ziekenhuis o Werk o Buiten o Andere plaats | core |
|  | EORTC QLQ-C30 / Facit-Pal |  | Context: Locatie | (Als thuis, bij iemand anders of ziekenhuis) Ik bevind me in bed. ¨ Ja ¨ Nee | core | Context: Locatie | *Indien thuis, bij iemand anders of ziekenhuis:* Ik bevind me in bed of zetel. | core |
|  | ESM Item Repository |  | Context: Locatie | Ik zou liever ergens anders zijn. | core | Context: Locatie | Ik ben tevreden met de plaats waar ik me nu bevind. | core |
|  | ESM Item Repository |  | Context: Medicatie | Sinds vorige biep heb ik het volgende gebruikt: ¨ Medicatie ¨ Sigaretten (incl. elektronische) ¨ Alcohol ¨ Caffeïne ¨ Niets ¨ Andere middelen: _____________________ | core | Context: Middelen | Sinds vorige biep heb ik het volgende gebruikt:  o Medicatie o Sigaretten o Alcohol o Cafeïne (bv. koffie) o Niets o Andere middelen | core |
|  |  |  | Context: Medicatie | Indien medicatie gebruikt, Ik heb medicatie genomen tegen: ¨ Pijn ¨ Misselijkheid ¨ Anderen: … | core | Context: Middelen | Indien medicatie gebruikt: Ik heb medicatie genomen tegen:  o Pijn o Misselijkheid o Angst of onrust o Anderen | core |
| Lichamelijke symptomen | EORTC QLQ-C30 / Facit-Pal / IPOS | Op dit moment heb ik pijn. | Lichamelijke klachten | Op dit moment heb ik pijn. | core | Lichamelijke klachten | Op dit moment heb ik pijn. | core |
| / |  |  | Lichamelijke klachten | Indien pijn: De pijn bevindt zich aan deze lichaamsdelen [duid de plaats(en) van de pijn aan op het popje]. | core | Lichamelijke klachten | Indien pijn: De pijn bevindt zich aan deze lichaamsdelen. | core |
| Lichamelijke symptomen | EORTC QLQ-C30 / Facit-Pal / IPOS | Op dit moment ben ik kortademig. | Lichamelijke klachten | Op dit moment ervaar ik ademhalingsproblemen (kortademigheid, moeilijk adem kunnen krijgen). | core | Lichamelijke klachten | Op dit moment ervaar ik ademhalingsproblemen (kortademigheid, moeilijk adem kunnen krijgen). | core |
| Lichamelijke symptomen | EORTC QLQ-C30 / Facit-Pal / IPOS | Op dit moment voel ik me misselijk. | Lichamelijke klachten | Op dit moment voel ik me misselijk. | core | Lichamelijke klachten | Op dit moment voel ik me misselijk. | core |
| Lichamelijke symptomen | EORTC QLQ-C30 | Op dit moment voel ik me moe. | Lichamelijke klachten | Op dit moment voel ik me moe. | core | Lichamelijke klachten | Op dit moment voel ik me moe. | core |
|  | ESM Item Repository |  | Meta | Ik vind het storend om nu de vragenlijst in te vullen. | core | Meta | Ik vind het storend om nu deze vragenlijst in te vullen. | core |
|  | ESM Item Repository |  | Meta | Het kostte me moeite om deze vragenlijst in te vullen. | core | Meta | Het kost me moeite om deze vragenlijst in te vullen. | core |
|  | ESM Item Repository |  | Meta | Ik heb de vragen aandachtig ingevuld. | core | Meta | Ik heb de vragen aandachtig ingevuld. | core |
|  |  |  | Negatieve gevoelens | Op dit moment voel ik me onrustig. | core | Negatieve gevoelens | Op dit moment voel ik me onrustig. | core |
| Negatieve gevoelens | Facit-Pal / ESM Item Repository | Op dit moment voel ik me verdrietig. | Negatieve gevoelens | Op dit moment voel ik me verdrietig. | core | Negatieve gevoelens | Op dit moment voel ik me verdrietig. | core |
| Positieve gevoelens | ESM Item Repository | Op dit moment voel ik me tevreden. | Positieve gevoelens | Op dit moment voel ik me tevreden. | core | Positieve gevoelens | Op dit moment voel ik me tevreden. | core |
| Positieve gevoelens | ESM Item Repository | Op dit moment voel ik me ontspannen. | Positieve gevoelens | Op dit moment voel ik me ontspannen. | core | Positieve gevoelens | Op dit moment voel ik me ontspannen. | core |
| Problemen met het denken | EORTC QLQ-C30 | Sinds vorige biep had ik moeite met het concentreren op dingen (zoals de krant lezen of televisie kijken). | Problemen met het denken | Sinds vorige biep had ik moeite met het concentreren op dingen zoals de krant lezen, televisie kijken of een gesprek volgen. | core | Problemen met het denken | Sinds vorige biep had ik moeite met het concentreren op dingen zoals de krant lezen, televisie kijken of een gesprek volgen. | core |
| Negatieve gevoelens | ESM Item Repository | Op dit moment voel ik me angstig. | Psychologisch welzijn | Op dit moment voel ik me angstig. | core | Psychologisch welzijn | Op dit moment voel ik me angstig. | core |
| Psychologische kwetsbaarheden | EORTC QLQ-C30 / Facit-Pal / IPOS | Op dit moment maak ik me zorgen. | Psychologisch welzijn | Op dit moment maak ik me zorgen. | core | Psychologisch welzijn | Op dit moment maak ik me zorgen. | core |
|  | IPOS / ESM Item Repository |  | Psychologisch welzijn | Op dit moment voel ik me depressief. | core | Psychologisch welzijn | Op dit moment voel ik me depressief. | core |
| Negatieve gevoelens | ESM Item Repository | Op dit moment voel ik me eenzaam. | Sociaal welzijn | Op dit moment voel ik me eenzaam. | core | Sociaal welzijn | Op dit moment voel ik me eenzaam. | core |
| Positieve gevoelens | ESM Item Repository / Facit-Pal / IPOS | Op dit moment voel ik me energiek. | Positieve gevoelens | Op dit moment voel ik me energiek. | core | Positieve gevoelens | Op dit moment voel ik me energiek. | core |
| Lichamelijk functioneren | EORTC QLQ-C30 / Facit-Pal | Sinds vorige biep heb ik moeite gehad met het doen van inspanningen zoals het dragen van een zware boodschappentas of een koffer. | Lichamelijk functioneren | Vandaag heb ik door mijn lichamelijke toestand moeite gehad met het uitoefenen van mijn dagelijkse bezigheden. | core: avond | Lichamelijk functioneren | Vandaag heb ik door mijn lichamelijke toestand moeite gehad met het uitoefenen van mijn dagelijkse bezigheden. | core: avond |
|  | ESM Item Repository |  | Meta | Vandaag heb ik bewust niet op een biep gereageerd. ¨ Ja ¨ Nee | core: avond | Meta | Vandaag heb ik bewust niet op een biep gereageerd. o Ja o Nee | core: avond |
|  | ESM Item Repository |  | Meta | Ik heb niet op die biep(s) gereageerd omdat: ¨ Ik kon niet (op tijd) reageren ¨ Ik was aan het slapen of rusten ¨ Ik had geen zin  ¨ Ik was te gestrest ¨ Ik ervaarde de biep als belastend ¨ Anders: _____________________________ | core: avond | Meta | Ik heb niet op die biep(s) gereageerd omdat: o Ik kon niet (op tijd) reageren o Ik was aan het slapen of rusten o Ik had geen zin o Ik was te gestrest o De vragenlijst zou me te veel tijd kosten o Ik ervaarde de biep als belastend o Anders | core: avond |
| Psychologische kwetsbaarheden | Facit-Pal | Op dit moment kan ik van het leven genieten. | Psychologisch welzijn | Ik heb het gevoel dat ik vandaag van het leven heb kunnen genieten. | core: avond | Psychologisch welzijn | Ik heb het gevoel dat ik vandaag van mijn dag heb kunnen genieten. | core: avond |
| Sociaal | Facit-Pal | Op dit moment voel ik morele steun van mijn naasten. | Sociaal welzijn | Vandaag voelde ik me gesteund door anderen. | core: avond | Sociaal welzijn | Vandaag kreeg ik van mijn naaste(n) de steun die ik nodig had. | core: avond |
| Sociaal | Facit-Pal | Op dit moment heb ik het gevoel dat ik mijn naasten tot last ben. | Sociaal welzijn | Vandaag had ik het gevoel dat ik mijn naasten tot last was. | core: avond | Sociaal welzijn | Vandaag had ik het gevoel dat ik mijn naaste(n) tot last was. | core: avond |
| Spiritueel en zingevend | Facit-Pal | Op dit moment ben ik hoopvol. | Spiritueel en zingevend welzijn | Vandaag voelde ik me hoopvol. | core: avond | Spiritueel en zingevend welzijn | Vandaag voelde ik me hoopvol. | core: avond |
| Slaapkwaliteit | EORTC QLQ-C30 / Facit-Pal | Deze nacht heb ik goed geslapen. | Slaapkwaliteit | Deze nacht heb ik goed geslapen. | core: morning | Slaapkwaliteit | Deze nacht heb ik goed geslapen. | core: morning |
|  |  |  | Slaapkwaliteit | Indien niet goed geslapen: Ik denk dat ik minder goed sliep, omdat: | core: morning | Slaapkwaliteit | Indien niet goed geslapen: Ik denk dat ik minder goed sliep, omdat: | core: morning |
| Lichamelijke symptomen | EORTC QLQ-C30 / Facit-Pal / IPOS | Sinds vorige biep heb ik overgegeven. | Lichamelijke klachten | Sinds vorige biep heb ik overgegeven. | suppl | Lichamelijke klachten | Sinds vorige biep heb ik overgegeven. | suppl |
| Lichamelijke symptomen | EORTC QLQ-C30 / Facit-Pal / IPOS | Op dit moment heb ik last van verstopping van de darmen. | Lichamelijke klachten | Op dit moment heb ik last van verstopping van de darmen. | suppl | Lichamelijke klachten | Op dit moment heb ik last van verstopping van de darmen. | suppl |
| Lichamelijke symptomen | EORTC QLQ-C30 | Op dit moment voel ik me slap. | Lichamelijke klachten | Op dit moment voel ik me slap. | suppl | Lichamelijke klachten | Op dit moment voel ik me slap. | suppl |
|  |  |  | Lichamelijke klachten | Op dit moment heb ik last van neuropathie (bv. tintelingen of pijn). | suppl | Lichamelijke klachten | Vandaag had ik last van zenuwschade (bv. tintelingen, voos gevoel of pijn). | suppl |
|  |  |  | Lichamelijke klachten | Indien last van neuropathie: De neuropathie die ik ervaar bevindt zich: | suppl | Lichamelijke klachten | *Indien zenuwschade > 10:* De zenuwschade die ik vandaag ervaarde bevond zich: | suppl |
| Lichamelijke symptomen | FACIT-Pal / IPOS | Op dit moment heb ik een droge mond en keel. | Lichamelijke klachten | Op dit moment heb ik een droge mond en keel. | suppl | Lichamelijke klachten | Op dit moment heb ik een droge mond of keel. | suppl |
| Lichamelijke symptomen | EORTC QLQ-C30 | Sinds vorige biep heb ik diarree gehad. | Lichamelijke klachten | Sinds vorige biep heb ik diarree gehad. | suppl | Lichamelijke klachten | Sinds vorige biep heb ik diarree gehad. | suppl |
| Lichamelijke symptomen | EORTC QLQ-C30 | Op dit moment heb ik behoefte om te rusten. | Lichamelijke klachten | Op dit moment heb ik behoefte om te rusten. | suppl | Lichamelijke klachten | Op dit moment heb ik behoefte om te rusten. | suppl |
| Negatieve gevoelens | Facit-Pal / ESM Item Repository | Op dit moment voel ik me nerveus. | Negatieve gevoelens | Op dit moment voel ik me nerveus. | suppl | Negatieve gevoelens | Op dit moment voel ik me nerveus. | suppl |
| Negatieve gevoelens | EORTC QLQ-C30 / ESM Item Repository | Op dit moment voel ik me gespannen. | Negatieve gevoelens | Op dit moment voel ik me gespannen. | suppl | Negatieve gevoelens | Op dit moment voel ik me gespannen. | suppl |
| Negatieve gevoelens | ESM Item Repository | Op dit moment voel ik me gestrest. | Negatieve gevoelens | Op dit moment voel ik me gestrest. | suppl | Negatieve gevoelens | Op dit moment voel ik me gestrest. | suppl |
| Negatieve gevoelens | ESM Item Repository | Op dit moment voel ik me prikkelbaar. | Negatieve gevoelens | Op dit moment voel ik me prikkelbaar. | suppl | Negatieve gevoelens | Op dit moment voel ik me prikkelbaar. | suppl |
| Negatieve gevoelens | EORTC QLQ-C30 / ESM Item Repository | Op dit moment voel ik me neerslachtig. | Negatieve gevoelens | Op dit moment voel ik me neerslachtig. | suppl | Negatieve gevoelens | Op dit moment voel ik me neerslachtig. | suppl |
| Negatieve gevoelens | ESM Item Repository | Op dit moment voel ik me lusteloos. | Negatieve gevoelens | Op dit moment voel ik me lusteloos. | suppl | Negatieve gevoelens | Op dit moment voel ik me lusteloos. | suppl |
|  |  |  | Negatieve gevoelens | Op dit moment voel ik me kwaad. | suppl | Negatieve gevoelens | Op dit moment voel ik me kwaad. | suppl |
| Positieve gevoelens | ESM Item Repository | Op dit moment voel ik me opgewekt. | Positieve gevoelens | Op dit moment voel ik me vrolijk. | suppl | Positieve gevoelens | Op dit moment voel ik me vrolijk. | suppl |
| Positieve gevoelens | ESM Item Repository | Op dit moment voel ik me kalm. | Positieve gevoelens | Op dit moment voel ik me kalm. | suppl | Positieve gevoelens | Op dit moment voel ik me kalm. | suppl |
| Problemen met het denken | EORTC QLQ-C30 | Sinds vorige biep heb ik moeite gehad met het herinneren van dingen. | Problemen met het denken | Sinds vorige biep heb ik moeite gehad met het herinneren van dingen. | suppl | Problemen met het denken | Sinds vorige biep heb ik moeite gehad met het herinneren van dingen. | suppl |
|  |  |  | Professioneel leven | Op dit moment voel ik me capabel om te werken. | suppl | Professioneel leven | Op dit moment voel ik me capabel om te werken (thuiswerk inbegrepen). | suppl |
|  |  |  | Psychologisch welzijn | Op dit moment heb ik negatieve gedachten of gevoelens. | suppl | Psychologisch welzijn | Op dit moment heb ik negatieve gedachten of gevoelens. | suppl |
| Spiritueel en zingevend | EORTC QLQ-C30 | Op dit moment voel ik me onafhankelijk. | Lichamelijk functioneren | Vandaag heb ik alles kunnen doen wat ik wou doen. | suppl: avond | Lichamelijk functioneren | Ik ben tevreden met alles wat ik vandaag heb kunnen doen. | suppl: avond |
| Lichamelijke symptomen | EORTC QLQ-C30 / IPOS | Op dit moment heb ik gebrek aan eetlust. | Lichamelijke klachten | Op dit moment heb ik gebrek aan eetlust. | suppl: avond | Lichamelijke klachten | Vandaag had ik gebrek aan eetlust. | suppl: avond |
| Lichamelijke symptomen | FACIT-Pal | Op dit moment zijn sommige delen van mijn lichaam opgezwollen. | Lichamelijke klachten | Vandaag waren sommige delen van mijn lichaam opgezwollen. | suppl: avond | Lichamelijke klachten | Vandaag waren sommige van mijn lichaamsdelen opgezwollen. | suppl: avond |
|  | FACIT-Pal |  | Psychologisch welzijn | Vandaag heeft mijn werk (huishouden inbegrepen) mij voldoening gegeven. | suppl: avond | Psychologisch welzijn | Vandaag heeft mijn werk of huishouden mij voldoening gegeven. | suppl: avond |
|  |  |  | Sociaal welzijn | Vandaag heb ik me zorgen gemaakt over mijn naasten. | suppl: avond | Sociaal welzijn | Vandaag heb ik me zorgen gemaakt over mijn naasten. | suppl: avond |
|  | EORTC QLQ-C30 |  | Sociaal welzijn | Vandaag heeft mijn lichamelijke toestand of behandeling mijn familieleven in de weg gestaan. | suppl: avond | Sociaal welzijn | Vandaag heeft mijn lichamelijke toestand of behandeling mijn familieleven in de weg gestaan (bv. plannen met gezin moeten aanpassen). | suppl: avond |
|  | EORTC QLQ-C30 |  | Sociaal welzijn | Vandaag heeft mijn lichamelijke toestand of behandeling mij belemmerd bij mijn sociale bezigheden. | suppl: avond | Sociaal welzijn | Vandaag heeft mijn lichamelijke toestand of behandeling mij belemmerd bij mijn sociale bezigheden (bv. plannen met vrienden moeten aanpassen). | suppl: avond |
|  | FACIT-Pal |  | Sociaal welzijn | Vandaag heb ik mijn zorgen openlijk kunnen bespreken met mijn naasten. | suppl: avond | Sociaal welzijn | Ik heb vandaag mijn zorgen besproken met mijn naasten. - Indien ja: Ik ben tevreden over de manier waarop ik mijn zorgen heb kunnen bespreken met mijn naaste(n). - Indien nee: Ik had graag mijn zorgen wel openlijk kunnen bespreken met mijn naaste(n). | suppl: avond |
|  | FACIT-Pal |  | Sociaal welzijn | Vandaag was ik tevreden met de communicatie over mijn ziekte met mijn naasten. | suppl: avond | Sociaal welzijn | Vandaag was ik tevreden met de communicatie over mijn ziekte met mijn naasten. | suppl: avond |
|  | FACIT-Pal |  | Sociaal welzijn | Vandaag had ik het gevoel dat mijn familie mij waardeert. | suppl: avond | Sociaal welzijn | Vandaag had ik het gevoel dat mijn familie mij waardeert. | suppl: avond |
|  | IPOS |  | Sociaal welzijn | Vandaag heeft iemand van mijn familie of vrienden zich angstig of bezorgd gevoeld over mij. | suppl: avond | Sociaal welzijn | Vandaag heeft iemand van mijn familie of vrienden zich angstig of bezorgd gevoeld over mij. | suppl: avond |
| Spiritueel en zingevend | FACIT-Pal | Op dit moment voel ik me nuttig. | Spiritueel en zingevend welzijn | Vandaag heb ik me nuttig gevoeld. | suppl: avond | Spiritueel en zingevend welzijn | Vandaag heb ik me nuttig gevoeld. | suppl: avond |
|  |  |  |  | Vandaag heb ik me verbonden gevoeld met mijn geloof. | suppl: avond | Spiritueel en zingevend welzijn | Vandaag heb ik me verbonden gevoeld met mijn geloof. | suppl: avond |
|  |  |  |  | Vandaag vond ik het leven zinvol. | suppl: avond | Spiritueel en zingevend welzijn | Vandaag vond ik het leven zinvol. | suppl: avond |
|  | FACIT-Pal |  |  | Vandaag heb ik zoveel mogelijk uit mijn dag gehaald. | suppl: avond | Spiritueel en zingevend welzijn | Vandaag heb ik zoveel mogelijk uit mijn dag gehaald. | suppl: avond |
|  |  |  |  |  |  | Lichamelijke klachten | Sinds de vorige biep heb ik last gehad van spierkrampen. | suppl |
|  |  |  | Slaapkwaliteit | De voorbije nacht ben ik … keer opgestaan. | suppl: morning | Slaapkwaliteit | De voorbije nacht ben ik … keer opgestaan. o 0 o 1-2 o 3-4 o Meer dan 5 | suppl: morning |
|  |  |  | Slaapkwaliteit | De voorbije nacht had ik problemen met terug in te slapen na het opstaan. | suppl: morning | Slaapkwaliteit | De voorbije nacht had ik problemen met terug in te slapen na het opstaan. | suppl: morning |
|  |  |  | Sociaal welzijn | Ik ben tevreden over de mate waarin ik gisteren intimiteit ervaren heb. | suppl: morning | Sociaal welzijn | Ik ben tevreden over de mate waarin ik gisteren intimiteit met mijn partner ervaren heb. | suppl: morning |
|  | EORTC QLQ-C30 |  | Context: Activiteiten | Deze activiteit is nu moeilijk voor mij. | core |  |  |  |
|  | ESM Item Repository |  | Context: Gebeurtenis | Denk aan de meest plezierige gebeurtenis of activiteit sinds de vorige biep. Hoe plezierig was dit? | core |  |  |  |
|  | ESM Item Repository |  | Context: Gebeurtenis | De plezierige gebeurtenis had te maken met… | core |  |  |  |
|  | ESM Item Repository |  | Context: Gebeurtenis | Denk aan de meest onplezierige gebeurtenis of activiteit sinds de vorige biep. Hoe onplezierig was dit? | core |  |  |  |
|  | ESM Item Repository |  | Context: Gebeurtenis | De onplezierige gebeurtenis had te maken met… | core |  |  |  |
|  | ESM Item Repository |  | Meta | Denk terug aan wat je aan het doen was net voor de biep. Gelieve voor deze vraag <Helemaal niet> te kiezen. | core |  |  |  |
| Lichamelijke symptomen | FACIT-Pal / IPOS | Op dit moment ontbreekt het me aan energie. |  |  |  |  |  |  |
| Lichamelijk functioneren | EORTC QLQ-C30 | Sinds vorige biep heb ik hulp nodig gehad met eten, aankleden, mezelf wassen of naar het toilet gaan. |  |  |  |  |  |  |
| Lichamelijk functioneren | EORTC QLQ-C30 | Sinds vorige biep ben ik beperkt geweest bij het uitoefenen van mijn hobby of bij andere bezigheden die ik in mijn vrije tijd doe. |  |  |  |  |  |  |
| Lichamelijk functioneren | EORTC QLQ-C30 | *(Als pijn)* Heeft deze pijn u sinds vorige biep gehinderd bij uw dagelijkse bezigheden? |  |  |  |  |  |  |
| Lichamelijk functioneren | FACIT-Pal | Sinds vorige biep heb ik door mijn lichamelijke toestand moeite gehad om aan de behoefte van mijn gezin/familie tegemoet te komen. |  |  |  |  |  |  |
| Lichamelijk functioneren | IPOS | Sinds vorige biep heb ik last gehad van verminderde mobiliteit. |  |  |  |  |  |  |
| Lichamelijke symptomen | FACIT-Pal | Op dit moment heb ik last van de bijwerkingen van de behandelingen. |  |  |  |  |  |  |
| Lichamelijke symptomen | FACIT-Pal | Op dit moment voel ik me ziek. |  |  |  |  |  |  |
| Lichamelijke symptomen | IPOS | Op dit moment voel ik me slaperig. |  |  |  |  |  |  |
| Negatieve gevoelens | ESM Item Repository | Op dit moment voel ik me geïrriteerd. |  |  |  |  |  |  |
| Negatieve gevoelens | ESM Item Repository | Op dit moment voel ik me somber. |  |  |  |  |  |  |
| Positieve gevoelens | ESM Item Repository | Op dit moment voel ik me enthousiast. |  |  |  |  |  |  |
| Problemen met het denken | FACIT-Pal | Op dit moment heb ik het gevoel dat ik helder kan denken. |  |  |  |  |  |  |
| Psychologische kwetsbaarheden | IPOS | Op dit moment ervaar ik innerlijke rust. |  |  |  |  |  |  |
| Psychologische kwetsbaarheden | FACIT-Pal | Op dit moment ben ik tevreden over hoe ik met mijn ziekte omga. |  |  |  |  |  |  |
| Spiritueel en zingevend | FACIT-Pal | Op dit moment ben ik in staat om beslissingen te nemen. |  |  |  |  |  |  |
| Spiritueel en zingevend | FACIT-Pal | Op dit moment aanvaard ik mijn ziekte. |  |  |  |  |  |  |
