# Supplementary material for: Uncovering the Daily Experiences of People Living With Advanced Cancer Using an Experience Sampling Method Questionnaire: Development, Content Validation, and Optimization Study
Source: JMIR Cancer. 2024 Nov 5;10:e57510. doi: 10.2196/57510 (PMC11576598; doi:10.2196/57510)
Supplement: Multimedia Appendix 7 [file cancer_v10i1e57510_app7.docx]

**Multimedia Appendix 7.** Final core ESM-AC^a^ questionnaire.

| Assessment schedule, subdomain, and item | | | | Response options |
| --- | --- | --- | --- | --- |
| **Momentary** | | | | |
|  | | **Physical symptoms** | | |
|  |  | | 1. “At this moment, I have pain.” | - Slider: 0=not at all and 100=very much |
|  |  | | 1a. If item 1 scored >10*:* “The pain is located at these body parts.” | - Multiple choices - Head - Back - Hands or fingers - Stomach - Hips - Knees - Feet or toes - Other body parts |
|  |  | | 2. “At this moment, I feel tired.” | - Slider: 0=not at all and 100=very much |
|  |  | | 3. “At this moment, I feel nauseated.” | - Slider: 0=not at all and 100=very much |
|  |  | | 4. “At this moment, I’m experiencing breathing problems (shortness of breath or difficulty breathing).” | - Slider: 0=not at all and 100=very much |
|  | | **Negative affect** | | |
|  |  | | 5. “At this moment, I feel restless.” | - Slider: 0=not at all and 100=very much |
|  |  | | 6. “At this moment, I feel sad.” | - Slider: 0=not at all and 100=very much |
|  | | **Positive affect** | | |
|  |  | | 7. “At this moment, I feel content.” | - Slider: 0=not at all and 100=very much |
|  |  | | 8. “At this moment, I feel relaxed.” | - Slider: 0=not at all and 100=very much |
|  |  | | 9. “At this moment, I feel energized.” | - Slider: 0=not at all and 100=very much |
|  | | **Cognitive complaints** | | |
|  |  | | 10. “Since last beep, I had trouble concentrating on things like reading a newspaper, watching television, or following a conversation.” | - Slider: 0=not at all and 100=very much |
|  | | **Psychological well-being** | | |
|  |  | | 11. “At this moment, I feel worried.” | - Slider: 0=not at all and 100=very much |
|  |  | | 12. “At this moment, I feel depressed.” | - Slider: 0=not at all and 100=very much |
|  |  | | 13. “At this moment, I feel anxious.” | - Slider: 0=not at all and 100=very much |
|  | | **Social well-being** | | |
|  |  | | 14. “At this moment, I feel lonely.” | - Slider: 0=not at all and 100=very much |
|  | | **Global well-being** | | |
|  |  | | 15. “At this moment, I feel...” | - Slider: 0=very bad and 100=very good |
|  |  | | 16. “If there is anything else you want to note about the period since last beep, you can do it here:” | - Open question |
|  | | **Social company** | | |
|  |  | | 17. “Who was with me at the moment of the beep?” | - Multiple choices - Partner - Child(ren) - Other family members - Friend(s) - Acquaintance(s) - Health care provider - Coworker(s) - Online contact (such as WhatsApp) or phone call - Others - Nobody (I am alone) |
|  | | **Social company (Appraisal)** | | |
|  |  | | 18a. If not “Nobody (I am alone)”: “I think this company is pleasant.” | - Slider: 0=not at all and 100=very much |
|  |  | | 18b. If “Nobody (I am alone)”: “It feels okay to be alone.” | - Slider: 0=not at all and 100=very much |
|  | | **Location** | | |
|  |  | | 19. “Where was I at the moment of the beep?” | - Multiple choices - At home - At someone else’s home - Store - Hospital - Work - Outside - Somewhere else |
|  | | **Location (appraisal)** | | |
|  |  | | 20. “I’m content with the place I was at.” | - Slider: 0=not at all and 100=very much |
|  | | **Location (bed/couch)** | | |
|  |  | | 21. If “At home,” “At someone else’s home,” or “Hospital”: *“*I was in bed or on the couch when the beep went off.” | - Yes or no |
|  | | **Activity** | | |
|  |  | | 22. “What was I doing at the moment of the beep?” | - Multiple choices - Active leisure (walking, cycling, odd jobs,...) - Passive leisure (watching television, internet, something quiet,...) - Work - Households, groceries, or home administration - En route (eg, on the bus) - Self-care or personal hygiene (washing, dressing...) - Eating or drinking - Taking care of my (grand)child - Conversation or interaction - Sleeping - Nothing - Something else |
|  | | **Activity (appraisal)** | | |
|  |  | | 23. If not “Nothing”: “I liked the activity I was doing right before the beep.” | - Slider: 0=not at all and 100=very much |
|  |  | | 24. If not “Nothing”: *“*I felt limited doing the activity right before the beep.” | - Slider: 0=not at all and 100=very much |
|  | | **Medication** | | |
|  |  | | 25. “Since last beep, I have used the following substance(s):” | - Multiple choices - Medication - Cigarettes - Alcohol - Caffeine (eg, coffee) - Nothing - Other substances |
|  |  | | 25a. If “Medication”: “I used medication against:” | - Multiple choices - Pain - Nausea - Anxiety or restlessness - Others |
|  | | **Meta (disturbance)** | | |
|  |  | | 26. “I thought it was disturbing to fill in this questionnaire” | - Slider: 0=not at all and 100=very much |
|  | | **Meta (difficulty)** | | |
|  |  | | 27. “It was difficult for me to complete this questionnaire.” | - Slider: 0=not at all and 100=very much |
|  | | **Meta (attention)** | | |
|  |  | | 28. “I completed the questions attentively.” | - Slider: 0=not at all and 100=very much |
| **Morning** | | | | |
|  | | **Sleep quality** | | |
|  |  | | 29. “This night, I slept well.” | - Slider: 0=not at all and 100=very much |
|  |  | | 29a. If item 29 scores >10*:* “I think I slept less well, because:” | - Open question |
| **Evening** | | | | |
|  | | **Physical functioning** | | |
|  |  | | 30. “Today, due to my physical condition, I had difficulty performing my daily activities.” | - Slider: 0=not at all and 100=very much |
|  | | **Psychological well-being** | | |
|  |  | | 31. “I feel like I was able to enjoy my day today.” | - Slider: 0=not at all and 100=very much |
|  | | **Social well-being** | | |
|  |  | | 32. “Today I received the support I needed from my loved one(s).” | - Slider: 0=not at all and 100=very much |
|  |  | | 33. “Today, I felt like I was a burden to my loved one(s).” | - Slider: 0=not at all and 100=very much |
|  | | **Spiritual-existential well-being** | | |
|  |  | | 34. “Today I felt hopeful.” | - Slider: 0=not at all and 100=very much |
|  | | **Meta (nonresponse)** | | |
|  |  | | 35. “Today I deliberately did not respond to a beep.” | - Yes or no |
|  |  | | 35. If “yes”: “I did not respond to that beep because:” | - Multiple choices - I could not react (on time). - I was sleeping or resting. - I did not feel like it. - I was too stressed. - The questionnaire would take me too much time. - I experienced the beep as burdensome. - Other |

^a^ESM-AC: Experience Sampling Method for People Living With Advanced Cancer.
