# Supplementary material for: Uncovering the Daily Experiences of People Living With Advanced Cancer Using an Experience Sampling Method Questionnaire: Development, Content Validation, and Optimization Study
Source: JMIR Cancer. 2024 Nov 5;10:e57510. doi: 10.2196/57510 (PMC11576598; doi:10.2196/57510)
Supplement: Multimedia Appendix 8 [file cancer_v10i1e57510_app8.docx]

**Multimedia Appendix 8.** Dutch onboarding session manual created after interview round three.

**Onboarding sessie**

Maak gebruik van een training sessie, analoog aan de gebruiksvriendelijkheid interviews, waarbij de patiënt eenmaal luidop de ESM vragenlijst invult en de nodige uitleg krijgt over de items, gevolgd door eenmaal de ESM vragenlijst invullen in stilte om te zien dat alles zonder problemen verloopt. Indien er zich nog grote problemen voordoen bij het invullen wordt de patiënt extra uitleg verschaft tot het mogelijk is om zonder problemen de ESM vragenlijst in te vullen.

**Doel van het onderzoek:**

- “We willen nagaan hoe mensen met kanker zich voelen en welke ervaringen ze mee maken in het dagelijks leven en hoe deze gevoelens en ervaringen veranderen doorheen de tijd, daarom bevragen we een aantal keer per dag dezelfde vragen voor een periode van 6 dagen”

**BASELINE VRAGENLIJST INVULLEN**

**EERSTE KEER INVULLEN: LUIDOP**

*Vraag tijdens de sessie eens om het toestel te ontgrendelen. Herhaal wanneer de patiënt hier wat meer moeite mee heeft.*

*Wanneer de patiënt onbewust de antwoordschaal omdraait en zo een antwoord geeft dat niet overkomt met wat hij/zij eigenlijk bedoelt: maak hen hiervan bewust en herhaal de vraag*

**Op dit moment voel ik pijn.** (eerste slider item)

- Deze schaal noemen we een slider (wat schuiver betekent). Wanneer je *op* deze schaal drukt zal er een bolletje verschijnen op de plaats waar je de schaal aanraakte. Je kan het bolletje verslepen. De positie van dit bolletje hoort overeen te komen met de sterkte van je gevoel dat je had op het moment dat de biep tijdens de studie afging.
- De waarde die boven de slider schaal komt te staan drukt de sterkte van jouw gevoel uit tussen 0 en 100, waarbij 0 vaak overeenkomt met “helemaal niet” en 100 vaak met “heel erg” (zoals te zien aan de woorden op de uitersten van de schaal). De waarde die er komt te staan hoeft maar een schatting te zijn, want het kan moeilijk zijn om één exacte waarde op je gevoel te plakken en bovendien ook moeilijk om op een rond getal te staan.
- Zoals je kan zien staat er verwoord: “op dit moment”. Hiermee bedoelen we dus effectief hoe je je op dit moment voelt, en dus niet gemiddeld gezien of hoe je je normaal op andere momenten voelt. Wanneer je het toestel mee naar huis krijgt zal je biepjes ontvangen die aangeven dat je de vragenlijst hoort in te vullen. Op dit momenten vul je dan de vragen in over het gevoel dat je had op het moment dat de biep afging.

**De pijn bevindt zich aan deze lichaamsdelen:** (eerste multiple choice item, indien de patient pijn ervaart)

- Indien het lichaamsdeel niet specifiek genoeg is aangeduid in deze lijst van antwoordopties kan u “andere lichaamsdelen” selecteren en nadien zelf de plaats van de pijn invullen invullen
- Zoals je kan zien staan er vinkjes voor de antwoordopties, dit wilt zeggen dat je meerdere opties tegelijk kan selecteren indien nodig.

**Sinds vorige biep heb ik moeite gehad met het concentreren op dingen zoals de krant lezen, televisie kijken of een gesprek volgen.**

- Indien de patiënt de vraag interpreteert als bredere cognitie: Zoals je kan zien gaat deze vraag over je concentratie of aandacht sinds de vorige biep. Dit gaat enkel over problemen met je aandacht ergens op de houden en dus bijvoorbeeld niet over andere problemen zoals moeite met dingen herinneren.

**Op dit moment voel ik me …**

- Zoals je ziet bedoelen we met deze drie puntjes deze drie woorden onder de schaal, dus ofwel “heel slecht”, “neutraal” of “heel goed”, ofwel een gevoel ertussen. Dit is dus een beetje anders dan de andere slider schalen die gaan van “helemaal niet” tot “heel erg”, maar werkt wel met hetzelfde principe dat je erover mag schuiven met je vinger om de sterkte en richting van je gevoel aan te duiden

**Als u nog iets over de periode sinds vorige biep wilt noteren, dan kunt u dat hier doen:** (eerste open vraag)

- Dit is een andere soort vraag, namelijk een open vraag, je ziet hier een wit vak staan. Dat is een tekst vak. Als je er op drukt zal er een toetsenbord verschijnen waarmee je extra zaken kan typen die je wenst te vermelden. Als je het toetsenbord wilt laten verdwijnen druk je ergens buiten de tekst balk.
- Het is ook mogelijk om dit soort open vragen over te slaan. Dit kan je door onderaan de “volgende vraag”-knop op de blauwe “vraag overslaan” lettertjes te drukken. Dit kan enkel wanneer het een vraag is met een tekst balkje waar je zelf iets kan typen. Als je wel iets te vermelden hebt wordt je aangeraden om het wél te doen, zo komen we meer te weten over hoe je je op dat moment voelt.
- Dit “vraag overslaan” knopje is redelijk klein, dus het kan zijn dat je een beetje moet opletten tijdens het drukken hierop.

**Wie is er op dit moment bij mij aanwezig?** (eerste context vraag)

- Zoals je kan zien staat er verwoord: “op dit moment”. Hiermee bedoelen we dus effectief de situatie waarin je je bevond op het moment toen de biep afging. In dit geval dus wie er op dit moment bij jou in de buurt is.
- Zoals je kan zien is “anderen” een antwoordoptie hier. Wanneer er andere mensen bij jou in de buurt zijn op het moment van de biep, die niet tussen deze lijst staan, dan mag je de optie “anderen” gebruiken. Bijvoorbeeld nu ik als onderzoeker hier bij jou zit.
- Waarom we context en appraisals bevragen: Hierbij willen we ook vermelden dat we dus ook geïnteresseerd zijn in de situaties waarin mensen zich bevinden, maar ook hoe mensen zich voelen ten opzichte van die situaties, omdat dat deel uitmaakt van hoe mensen zich voelen tijdens hun dagelijks leven.

**Ik bevond me in bed of zetel toen de biep afging.**

- Bij deze vraag willen we dus weten of u zich op het moment toen de beep afging in bed of zetel bevond. Een stoel telt hier als “nee”. Als u in de zetel gaat zitten nadat de beep afging dan is het antwoord ook “nee”. Het gaat dus echt om het moment toen de beep afging.

**Wat deed ik toen de biep afging?**

- Zoals je kan zien gaat deze vraag over de activiteit die je aan het doen was net voor de biep afging. Dus elke keer wanneer je deze vraag krijgt in de loop van de komende week hoor je de activiteit te selecteren die je *op het moment van de biep* aan het doen was. Dus echt wanneer het geluidje van de smartphone afging en niet minuten ervoor.
- Zoals je kan zien staan er vinkjes voor de antwoordopties, wat wilt zeggen dat je meerdere activiteiten tegelijk kan selecteren indien nodig.

**Sinds vorige biep heb ik de volgende middelen gebruikt:**

- Wat denkt u dat we met deze middelen bedoelen? – we bevragen hiermee eigenlijk medicatie of andere verdovende of oppeppende middelen. Koffie valt daar bijvoorbeeld ook onder, via “Cafeïne”. Maar voedsel of water hoort hier dan weer niet onder. Dus snoep met veel suiker hoort hier bijvoorbeeld niet bij.
- Indien je andere verdovende of stimulerende middelen hebt geconsumeerd kan je die via de “andere middelen” aangeven.

**EINDE EERSTE KEER INVULLEN**

[Na de eerste keer invullen kan het voor de patiënt duidelijk zijn dat er ervaringen ontbreken die wel relevant zijn voor hem/haar. Daarom kan er op dit moment de supplementaire vragenlijst overlopen worden om te kijken of er 1 tot 2 items bij de core vragenlijst kunnen gevoegd worden.]

**TWEEDE KEER INVULLEN: IN STILTE**

*Geef verduidelijking wanneer de patiënt moeite heeft met iets in te vullen.*

**EINDE TWEEDE KEER INVULLEN**

De patiënt zou op dit punt probleemloos de vragenlijst moeten kunnen invullen. Indien dit niet het geval is kan de onderzoeker de vragen herhalen die een probleempunt vormden.

**Verwachting voor de studieperiode:**

- In de periode van 6 dagen die nu volgen zal je 10 beeps per dag horen. Wanneer er je een beep hoort is het de bedoeling dat je zo snel mogelijk de vragenlijst in vult, want we zijn benieuwd naar hoe je je voelde op het exact moment dat de beep afging.
- Er zijn natuurlijk wel geldige redenen om zo’n vragenlijst te missen, bijvoorbeeld als u met de wagen rijdt of aan het slapen bent. Op zulke momenten is het dus zeker oké om eens een vragenlijstje te missen.
- Hoewel je verwacht wordt om zo snel mogelijk na een beep de vragenlijst in te vullen voorzien we wel een korte tijd dat de vragenlijst beschikbaar blijft, bijvoorbeeld als je jezelf aan het wassen bent op het moment van de beep kan je eventjes later de vragenlijst nog invullen. Let wel op: we zijn dan nog steeds geïnteresseerd in hoe je je voelde op het moment toen de beep afging.
- Als u overdag wenst te slapen kan u het toestel in een andere ruimte leggen. Wanneer je klaar bent met slapen kan je dan het toestel er weer bijhalen.

ANDERE INSTRUCTIES:

- Oplader uitleggen
- Vragen om geregeld op te laden (best geen hele nachten want slecht voor batterij)
- Er is een ochtend en avond vragenlijst
- U zal opgebeld worden na 1-2 dagen om te kijken of alles nog duidelijk is
